# Genetic encoding of DNA nanostructures and their self-assembly in living bacteria

**DOI:** 10.1038/ncomms11179

**Published:** 2016-04-19

**Authors:** Johann Elbaz, Peng Yin, Christopher A. Voigt

**Affiliations:** 1 Synthetic Biology Center, Department of Biological Engineering, Massachusetts Institute of Technology, 500 Technology Square NE47-140, Cambridge, Massachusetts 02139, USA; 2 Wyss Institute for Biologically Inspired Engineering, Harvard University, Boston, Massachusetts 02115, USA; 3 Department of Systems Biology, Harvard Medical School, Boston, Massachusetts 02115, USA

## Abstract

The field of DNA nanotechnology has harnessed the programmability of DNA base pairing to direct single-stranded DNAs (ssDNAs) to assemble into desired 3D structures. Here, we show the ability to express ssDNAs in *Escherichia coli* (32–205 nt), which can form structures *in vivo* or be purified for *in vitro* assembly. Each ssDNA is encoded by a gene that is transcribed into non-coding RNA containing a 3′-hairpin (HTBS). HTBS recruits HIV reverse transcriptase, which nucleates DNA synthesis and is aided in elongation by murine leukemia reverse transcriptase. Purified ssDNA that is produced *in vivo* is used to assemble large 1D wires (300 nm) and 2D sheets (5.8 μm^2^) *in vitro*. Intracellular assembly is demonstrated using a four-ssDNA crossover nanostructure that recruits split YFP when properly assembled. Genetically encoding DNA nanostructures provides a route for their production as well as applications in living cells.

## Abstract (web summary)

DNA nanostructures have the potential to be powerful tools in many areas of biology however they are difficult to manufacture completely *in vivo*. Here the authors combine RNA hairpins and reverse transcription to generate and assemble a complex DNA structure inside the cellular environment.

## Graphical abstract

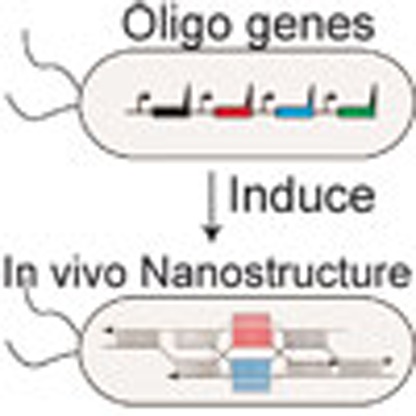

Since the early 1980s, it has been recognized that the information storage capacity of DNA is ideal for programming the self-assembly of nanostructures^1^. Different nucleotide sequences yield complementary strands that direct short single-stranded DNA (ssDNA) to hybridize with high specificity into a set of branched junctions, including the crossover^2^ and paranemic crossover^3^ motifs. These are the architectural elements that enable the self-assembly of larger 2D and three-dimensional (3D) nanostructures^4^,^5^,^6^. The structures that can be produced are incredibly intricate, including DNA origami (used to build a 100 nm 2D map of America)^7^ and DNA bricks (used to build a 25-nm 3D ‘spaceship')^8^. Software has simplified the process of designing oligos to assemble into user-defined structures^9^,^10^. The structures are not just static: dynamic DNA nanomachines have been built^11^,^12^, including walkers^13^,^14^,^15^, tweezers^16^,^17^ and gears^18^. Large computing circuits, including pattern-recognition algorithms, have been built based on catalytic nucleic acids (DNAzymes)^19^,^20^ and strand displacement^21^,^22^. Applications have been proposed, including scaffolds for composite materials^23^, catalysts^24^,^25^ and nanoparticles with controlled plasmonic properties^26^,^27^, intracellular sensors^28^ and drug delivery devices^29^,^30^,^31^.

The ability to create nanostructures within living cells using DNA has the potential to be a powerful tool for basic biology, biomedical engineering and medicine (Fig. 1a). Although it may seem counterintuitive that it is difficult to make DNA in cells, the production of short ssDNAs with precisely defined length and sequence has proven challenging. Clever techniques have been developed to make large quantities of oligos by encoding them in single-stranded phagemids, which are produced *in vivo*, cleaved *in vitro* using restriction enzymes and then performing the assembly reaction^32^,^33^,^34^. It has also been shown that ssDNA can be produced *in vivo* using a retron^35^. However, the desired nucleotides must be incorporated into a long DNA with complex secondary structure from which they would need to be cleaved as a second step. The ability of the ssDNAs to self-assemble into a desired nanostructure in the intracellular environment has not yet been demonstrated.

Here, we present a method that enables the short ssDNA to be encoded as a gene (r_oligo) that is expressed as a non-coding RNA (ncRNA) that is enzymatically converted to ssDNA. We demonstrate the use of the *in vivo* produced ssDNA for *in vitro* applications such as formation of one- (1D) and two-dimensional (2D) DNA wires and DNA sheets. Also, we demonstrate the ability to express and assemble DNA nanostructure within living cells. This is shown by building a four-ssDNA ‘crossover motif' that can act as a scaffold for proteins. This work offers both a route by which these structures could be made in bulk via biotechnology and to be induced in cells for *in vivo* applications.

## Results

### Design of the genetic part encoding ssDNA in bacteria

**Figure 1:**
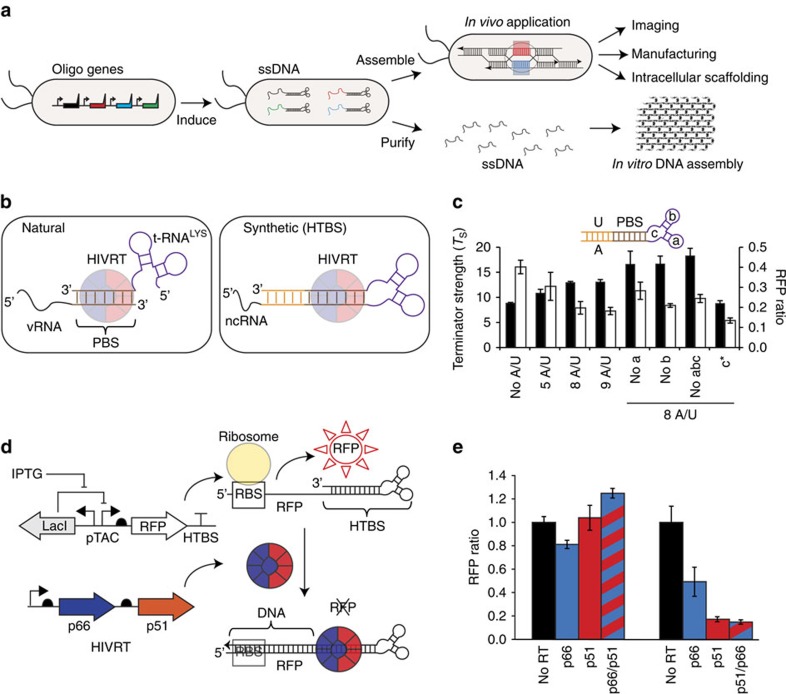
Genetic part design for *in vivo* ssDNA production. (**a**) The processes of ssDNA production and *in vitro*/*in vivo* assembly are shown. (**b**) The mechanism for the priming of HIVRT is compared for the natural system and the HTBS genetic part. HTBS, HIV terminator-binding site; HIVRT, HIV reverse transcriptase; ncRNA, non-coding RNA; PBS, protein-binding site; vRNA, viral RNA. (**c**) The terminator strength (black) and the RFP ratio (white) were measured for variations of HTBS parts. The terminator strengths were calculated as described previously^48^ by comparing the expression of two fluorescent reporters, one placed before and one after the HTBS part (for their plasmid map, see Supplementary Fig. 14). The knockdown in RFP expression corresponds to the experiments shown in **d**. To account for different baseline expression levels associated with terminator modifications, the RFP fluorescence generated by each of the different HTBSs is divided by the fluorescence measured in the absence of HIVRT (RFP ratio). Data shown represent the averages of three independent experiments performed on different days. For the different HTBS sequences, see Supplementary Table 1. (**d**) A schematic showing the use of HIVRT to knockdown gene expression is shown. (**e**) The impact of different combinations of the HIVRT subunits (columns coloured: black-no RT; p66-blue; p51-red and p66/p51-blue/red) on the RFP knockdown. The left sets of bars are a control containing a strong terminator (BBa_B0054) and the right sets of bars are for the HTBS. Data shown represent the averages of three independent experiments performed on different days.

The conversion of RNA into DNA is performed naturally by retroviruses, which have RNA genomes that need to be converted to DNA before integrating into the host genome^36^. The enzyme responsible is reverse transcriptase (RT), which has several roles, including functioning as a DNA- and RNA-dependent DNA polymerase, as an RNAase that cleaves the RNA from the DNA:RNA complex and to catalyse strand transfer and displacement synthesis^37^,^38^,^39^. The mechanism of RTs has been a subject of intensive research because it is a therapeutic target for HIV^40^ and is commonly used in molecular biology to quantify transcript abundance (RT–PCR)^41^. RTs have also been used *in vitro* as part of a DNA computing platform (RTRACS)^42^. Moreover, these eukaryotic retroviral RTs have been successfully expressed in bacteria and purified for *in vitro* experiments^43^ or used as a substitute for DNA polymerase I^44^. However, the possibility to functionally reverse transcribe RNA to DNA in bacteria using these eukaryotic retroviral RTs has not yet been shown. This may be due to the lack of eukaryotic t-RNA^LYS^, which is required for binding to the RT at the protein-binding site (PBS) and recruiting it to viral RNA (vRNA) to initiate polymerization (Fig. 1b)^45^,^46^.

It has been recognized that when t-RNA^LYS^ binds to the 3′ end of the vRNA the two molecules would create a single recognition RNA hairpin if the 3′-end of the vRNA were covalently attached to the 5′-end of the t-RNA (Fig. 1b; ref. 47). Thus, by designing a ncRNA to end with the recognition RNA hairpin, this may eliminate the need for a separate eukaryotic t-RNA^LYS^. Another advantage of fusing the recognition hairpin to the ncRNA is that the RT will only transcribe the desired RNA(s), thus eliminating the potential for crosstalk with free t-RNA^LYS^ and other intracellular RNAs. Implementing this requires that the recognition RNA hairpin also serves as a transcriptional terminator so that the ncRNA precisely ends after the PBS with the last nucleotide forming a basepair in order for HIVRT to begin DNA polymerization.

Using a mathematical model for guidance^48^, we hypothesized that the hairpin of the t-RNA could function as a transcriptional terminator in *E. coli*, which we confirmed experimentally (Fig. 1c). Various mutations were made to the hairpin that were predicted by the model and tested for increased termination strength (*T*_S_)^48^. For example, different poly-Us and their corresponding poly-As were placed, respectively, on both sides of the PBS and, as predicted, the termination strength increased in proportion to the number of poly-Us/poly-As added (Fig. 1c).

Next, we tested the ability for the recognition hairpins to recruit HIVRT when fused to the 3′-end of the ncRNA. To do this, an assay was developed based on the capability of HIVRT to block the translation of a targeted mRNA fused with the recognition hairpin (Fig. 1d; Methods). In the absence of HIVRT, the gene can be expressed. When HIVRT is expressed, polymerization of DNA on the targeted mRNA occurs and this blocks translation. This can be easily measured when the mRNA encodes red fluorescence protein (RFP), which reports the activity of HIVRT as a decrease in fluorescence. The hairpins were screened and the variant that contains a mutation close to a bulge (c*) and an additional 8 A/U bp was chosen (referred to as HIV Terminator-Binding Site, HTBS), which co-optimizes termination efficiency as well as the recruitment of HIVRT (Fig. 1c). Note that the 8 A/U bp are not part of the eukaryote's t-RNA^LYS^ nor the PBS and have been added to increase the termination efficiency.

HIVRT is a heterodimer composed of the p66 and p51 subunits^36^. The p66 subunit has three domains: a polymerase, a linker and an RNAse^38^,^39^,^40^. In the context of the virus, the p51 subunit is created by a post-translational mechanism, where the C-terminus of a p66/p66 homodimer is cleaved to remove the RNAse H domain. The p51 subunit contains a polymerase domain, but is mainly responsible for stabilizing the p66 subunit when bound to the vRNA^49^. Using the RFP assay, we tested for the requirement that both of the subunits be expressed, when they are encoded as separate genes and codon optimized for *E. coli* (Methods). In this assay, either subunit or both together are able to knockdown RFP expression (Fig. 1e).

### Production of ssDNA *in vivo*

**Figure 2:**
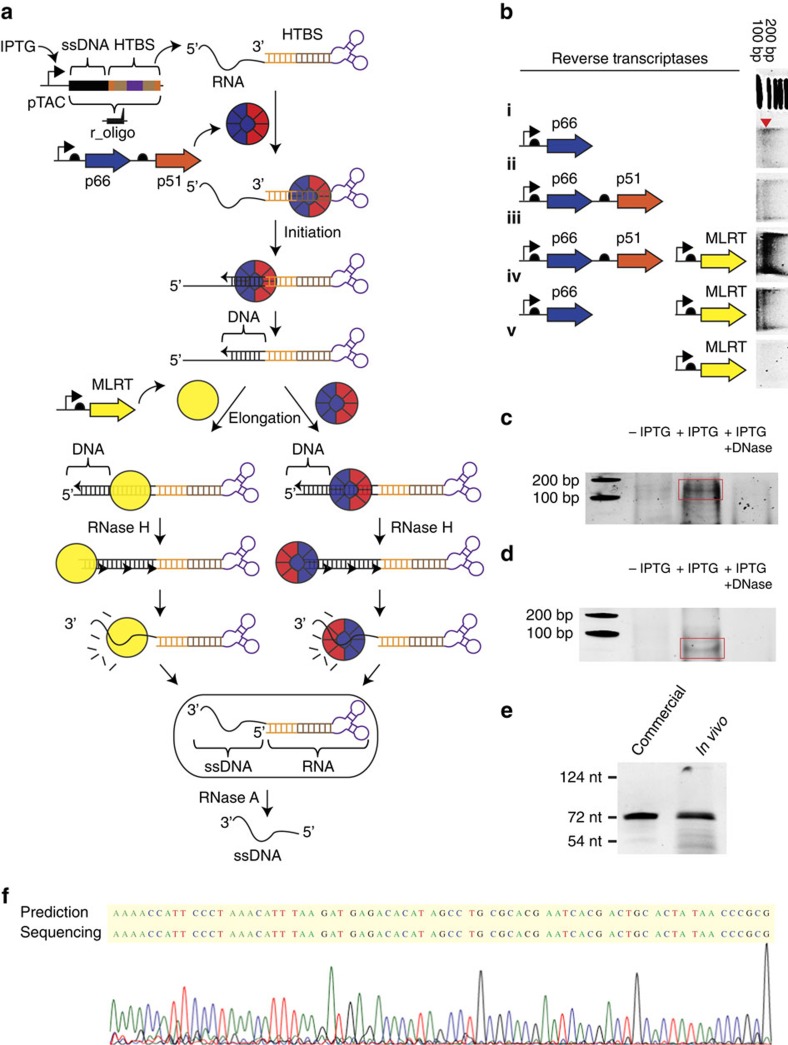
*In vivo* production of ssDNA. (**a**) Schematic for the conversion of the r_oligo gene to ssDNA. The r_oligo gene contains both the desired ssDNA sequence and the HTBS part, which serves as a terminator (black, ssDNA sequence; orange, A/U region; brown, PBS; purple, hairpin). (**b**) The impact of different combinations of RT expression on ssDNA production is shown. Data are shown for the production of a 205-nt ssDNA (r_oligo_205) under purification conditions preventing the removal of the HTBS (RNAse A+150 mM NaCl). The red triangle shows the predicted location of the band (note that the ladder is based on double stranded DNA). The bands are from the same gel and the image processed once, but the order changed for publication. For the full gel in its original order, see Supplementary Fig. 1. The ssDNA sequence and reverse transcriptase sequences are added in Supplementary Tables 2 and 3. (**c**) The expression of ssDNA after 18 h growth in the presence (+IPTG, 1 mM) and absence of IPTG (−IPTG) under conditions preserving the HTBS. To confirm that the band is ssDNA, the same sample is exposed to DNase (+IPTG/+DNase, 1 mM/4 units). (**d**) For the same system as in **c**, the ssDNA is treated to remove the HTBS RNA (RNAse and no salt). (**e**) Comparison of an *in vivo* produced ssDNA with commercial chemically synthesized ssDNAs. The ladder was calculated using commercial oligos of defined size run simultaneously in the gel. The ssDNA sequence is in Supplementary Table 2. (**f**) Sequencing analysis of the *in vivo* produced 72-mer. The ‘prediction' is the complementary sequence of the expected ssDNA (Methods).

The HIVRT subunits were then tested for the ability to produce ssDNA in cells (Fig. 2a). The r_oligo gene containing a 205-nt ssDNA sequence and HTBS is placed under pTAC control so that it can be induced with isopropyl-β-D-thiogalactoside (IPTG). We developed a purification protocol to isolate DNA products from lysed cells, which can be visualized using non-denaturing gel electrophoresis (Methods). All ssDNA production experiments are performed in the cloning strain *E. coli* DH10β, which lacks the intracellular exonuclease activity, thus preventing the degradation of ssDNA. This strain also lacks the SOS response, which could be induced by ssDNA. The expression of the p66 subunit alone is sufficient to observe a slight band at the correct length (Fig. 2b). The co-expression of the p51 subunit increases the production of the ssDNA because the p66/p51 complex has a higher affinity to the ncRNA substrate^49^. HIVRT is known to be slow as a DNA polymerase because it performs this function through multiple association and dissociation events and individual turnovers (versus a continuous progression)^50^,^51^. To increase production, we introduced a second RT from murine leukemia virus (MLRT), which is a DNA-dependent polymerase with strong RNAse H activity^52^. The MLRT gene is expressed under the control of a constitutive promoter from a separate plasmid (Methods). The expression of MLRT alone is unable to produce the ssDNA because of the HIVRT specificity of HTBS (Fig. 2b). When co-expressed with p66 or p66/p51, strong bands are observed. The expression of all three genes enhances the production of the ssDNA eightfold over p66 alone and threefold over both expressions of p66 and MLRT.

The r_oligo gene is under the control of the pTAC promoter; thus, it can be induced by IPTG and no ssDNA product is observed in the absence of inducer (Fig. 2c). This shows that the ssDNA requires r_oligo expression and is not a by-product of a nonspecific RT process. After purification, the HTBS motif was removed through the addition of RNase A in the absence of salt, leaving just the ssDNA (Fig. 2d). Treatment with DNase causes the band to disappear, confirming that it is a DNA product (Fig 2c,d). The ability to make diverse sequences of various length was demonstrated by producing 205, 72, 56 and 49 nt oligos (Fig. 2c–e and Supplementary Figs 1–4). The titre of the 72-nt oligo was calculated to be 4 μg l^−1^ (Methods).

The bands were compared with those obtained using commercial chemically synthesized ssDNA (Fig. 2e and Supplementary Fig. 3). To verify the sequence, the 72-nt band was polyacrylamide gel electrophoresis (PAGE) purified, recovered by PCR and sequenced by conjugating DNA adapters to the purified ssDNA (Fig. 2f and Supplementary Fig. 2)^53^. This method enables the sequencing of a short oligo and prevents the possibility of plasmid contamination (the DNA adapters can be ligated only to ssDNA and not to circular DNA). Further, we quantified the per-base error rate via deep sequencing to be 8.56 × 10^−4^. This is consistent with the published HIVRT error rate (5.9 × 10^−4^ to 5.3 × 10^−5^)^54^ and is lower than that obtained with chip-based oligo synthesis (2 × 10^−3^) (ref. 55).

### 1D and 2D DNA assemblies using *in vivo* ssDNA production

**Figure 3:**
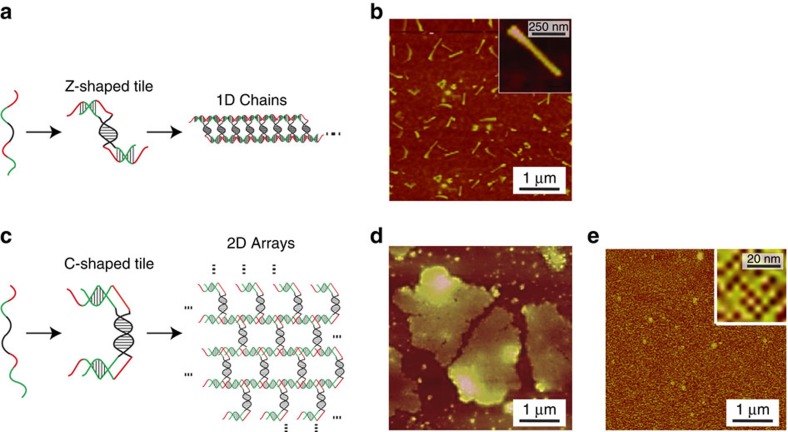
*In vitro* assembly of structures using *in vivo* produced ssDNA. (**a**) The mechanism for the 1D chain formation is shown. The colours on the ssDNA represent different functional domains (black, central palindrome; green, two flanking helical domains; red, T-junction tiles). The ssDNA sequence is added in Supplementary Table 2. (**b**) AFM images of the 1D chain. Insert: zoom-in. (**c**) The mechanism for the formation of 2D arrays is shown, from the self-assembly of a single ssDNA to a 2D DNA periodic arrays. The colours on the ssDNA represent different functional domains (black, a central palindrome; green, two flanking helical domains; red, T-junction tiles). The ssDNA sequence is added in Supplementary Table 2. (**d**) AFM images of the assembly of 2D arrays in solution are shown (Methods). (**e**) AFM images of the 2D arrays assemble using the surface-mediated assembly process (Methods). Inset: zoom-in. Larger-scale AFM images of the 1D chains and 2D arrays are shown in Supplementary Fig. 5.

We then purified ssDNAs produced *in vivo* and used them to form 1D DNA chains and 2D DNA arrays *in vitro*^56^. The formation of both structures is based on a periodic assembly method, where a single strand oligimerizes to form the larger structures (Fig. 3a,c). This ssDNA contains five domains: a central palindrome (black), two complementary segments (green) and two other complementary segments (red). First, two strands hybridize forming symmetric motifs through the homodimerization of the black domain and the hetrodimerization of the green domains while leaving the four red single-strand domains (C- and Z-shaped tiles). Then, two red pairs of a symmetric motif hybridize with two other red pairs from another symmetric motif forming a three-way junction leading to periodic assemblies. The size of the red domains is similar for both assemblies (a half-turn), while altering the lengths of the black domains from 1 to 1.5 turn helix and the green domains from half to one turn helix yields variations in the formation of different assemblies, 1D chains and 2D arrays, respectively.

We purified 1 μg of ssDNA produced *in vivo*, which is sufficient for the *in vitro* solution assembly methodology^56^. The resulting structures were analysed using tapping mode atomic force microscopy (AFM). The 1D structures have an average length of 300 nm (Fig. 3b) and the 2D arrays have an average area of 5.8 μm^2^ (Fig. 3d). Next, we used a surface-mediated assembly methodology^56^,^57^ to avoid the shear-induced breakage of the 2D arrays that enables the formation of unbroken 2D arrays and visualization of the internal structures (Fig. 3e). These results are comparable to the assemblies based on chemical ssDNA synthesis^56^. It is noteworthy that the ssDNAs in our system have been used directly after extraction from cell lysis and without PAGE or high-performance liquid chromatography purification.

### Production of DNA ‘crossover' nanostructures *in vivo*

**Figure 4:**
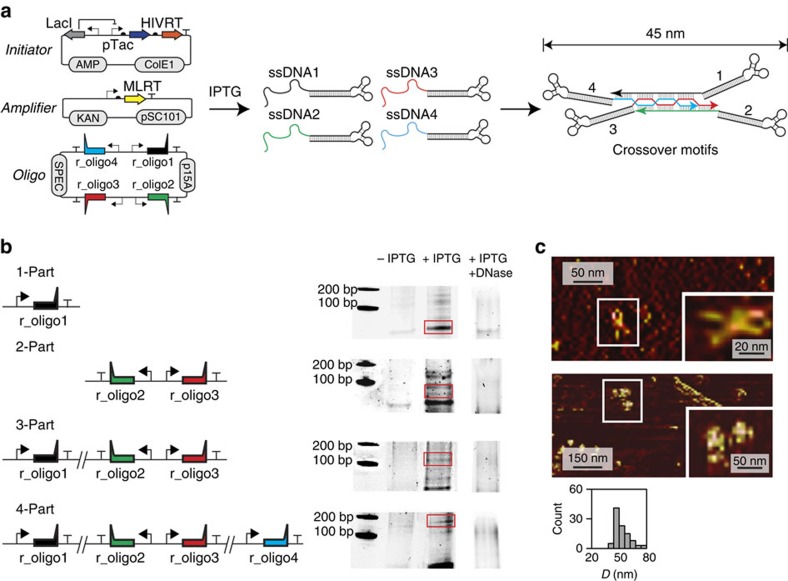
*In vivo* production of DNA nanostructures. (**a**) The three-plasmid production system and the structure of the 4-part DNA nanostructure are shown. The nanostructure contains eight crossover junctions distributed among the four strands. The size was calculated using NuPACK^70^. The ssDNA sequences are added in Supplementary Table 2. (**b**) The production of DNA nanostructures and its intermediate elements are shown when different combinations of r_oligo genes are expressed in the presence of IPTG (+IPTG, 1 mM) and the absence of IPTG (−IPTG) under conditions preserving the HTBS. The red bands show the approximate regions of the intermediate elements/DNA nanostructures (+IPTG/+DNase). Representative samples inducing the HIVRT and expressing the different r_oligo genes but exposed to DNase condition (the complete gel is shown in Supplementary Fig. 7). Insert: Zoom-in. (**c**) AFM images of the 4-part DNA nanostructure excised and purified from its appropriate band (as shown in **b**, the red square in the bottom gel). The size of the DNA nanostructure (*D*) in the form of a histogram is shown. The histogram has been calculated by auto-selecting all the particles with a minimum height of 0.5 nm and a maximum of 5 nm from the AFM images (Methods). For more AFM images, see Supplementary Fig. 8.

Next, we tested the ability to express and assemble DNA nanostructures *in vivo* (Fig. 4a). An initiator plasmid controls the expression of both genes for HIVRT under IPTG inducible control on a medium copy ColE1 origin. A second amplifier plasmid contains MLRT, which is constitutively expressed at low copy (psc101). Finally, all of the r_oligo genes are carried on a third p15a origin plasmid. Each gene is controlled with the same strong constitutive promoter (proD^58^) in order to keep the stoichiometry close to unity. The absolute concentrations and their ratios have been previously shown to be important for DNA assembly *in vitro*^4^,^5^,^6^. The selection of constitutive promoters of different strength would lead to different ratios of ssDNAs to be produced; the only requirement is that the +1 transcription start site be precise^59^ so that additional nucleotides do not appear on the 3′ end of the ssDNA. To reduce the potential impact of transcriptional read through, strong terminators (BBa_B0054) are placed after each r_oligo gene and they are encoded in alternating orientations.

Four ssDNAs were designed to assemble into a 45-nm nanostructure that is based on the crossover branched motif (Fig. 4a). This motif is a fundamental architectural unit core to many nanostructures^4^,^5^,^6^,^7^,^8^,^9^,^10^ representing different topologies and scales, ranging from 10 nm tetrahedra^5^ to 100 nm origami^7^. The motif is built using four 45-nt ssDNAs, each of which includes four 10-base sticky binding regions that connect the strands and form eight crossover junctions. The sequences of this region were selected based on the literature^2^ while modified by the addition of operators to which zinc finger domains will bind^60^. Additional changes were made to generate sequences that do not fold into undesirable secondary structures and assemblies (Methods). The remaining 5 nt part (TTTAT) at the 3′-end is added to eliminate the possibility of the RT from continuing to function as a polymerase on the DNA nanostructure by preventing the hybridization of the last 3′ base. The RNA hairpins were not cleaved in order to aid visualization by AFM and distinguish shapes associated with different combinations of oligos.

Different versions of the oligo plasmid were constructed to express 1, 2, 3 or all 4 ssDNAs (Fig. 4b). The ssDNAs were expressed and analysed using non-denaturing gel electrophoresis (Methods). In all cases, no bands are observed in the absence of IPTG (no HIVRT is expressed). When 1 mM IPTG is added, bands appear and their length shifts depending on how many ssDNAs are expressed. When ssDNA1 (45 nt) is expressed alone, the only base paired region is in the RNA hairpin (34 bp), and a strong band is observed at the correct length. When both ssDNA2 and ssDNA3 are expressed, this leads to several bands, including one at ∼90 bp. This shifts up to ∼110 bp when ssDNA1 is co-expressed with them. Finally, this shifts to ∼170 bp when all four ssDNAs are co-expressed. Note that the ssDNAs were only designed to form the complete four-part nanostructure. When only 2 or 3 are expressed, there are additional bands that form on the gel corresponding to alternate structures and these are almost eliminated when all four are expressed. We further performed the assembly of the DNA nanostructure using commercial chemically synthesized ssDNAs, and a similar effect on the DNA assembly was observed (Supplementary Fig. 6). Using a control experiment, we estimated that 90% of the material produced *in vivo* is lost during purification and recovery. Not accounting for this loss, the titres range from 7.5 μg l^−1^ when only ssDNA1 is expressed to 2 μg l^−1^ for the four-part crossover junction calculated based on spectroscopic absorbance measurements (Methods). Note that no ssDNA/nanostructure products are observed after DNAse treatment (Fig. 4b and Supplementary Fig. 7).

The four-part DNA nanostructure was purified and visualized using tapping AFM (Fig. 4c). The nick between the HTBS and the dsDNA allows for flexibility and this results in a ‘V' in the structure that simplifies the quantification of the final structure. The expression of all four ssDNAs forms ‘X' shaped structures and a size distribution with a peak at 45 nm. Note that no ‘X'-shaped structures were visualized via AFM after digestion of the HTBS. When the four-part system is expressed in the absence of the HIVRT gene, no bands were observed (Supplementary Fig. 9). From this gel, we further cut the region that would correspond to the four-part structure and visualized the product via AFM and, as expected, no structures were seen.

We also attempted to produce the 1D and 2D structures (Fig. 3) *in vivo*, but we were unable to see the assembled structures via AFM. This is consistent with the known conditions and timing required for the assembly of C- and Z-shaped tiles, which is not compatible with the intracellular environment^61^. Specifically, the 5- to 6-nt T-junctions may not form at physiological temperature, which prevents the extension of the periodic structures. This highlights that not all DNA nanostructures can be produced *in vivo* because of requirements in temperature, reaction conditions or timing that are not consistent with the intracellular environment.

### Intracellular sensor for the detection of DNA nanostructures

**Figure 5:**
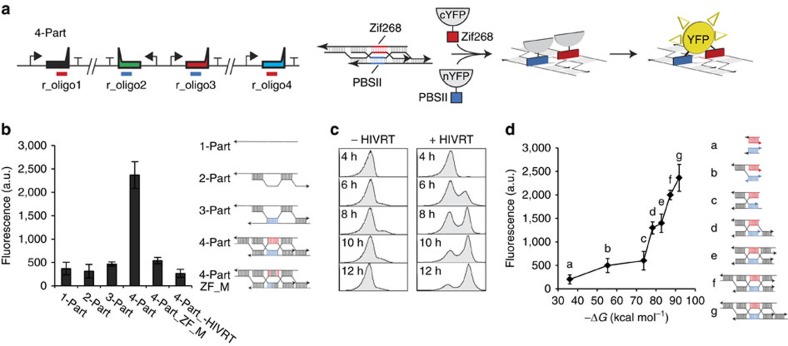
Intercellular DNA ‘crossover' nanostructure sensor. (**a**) A schematic of the *in vivo* sensor is shown. The zinc finger domains are underlined under each of the r_oligo genes and highlighted in the appropriate 10-bp domains formed by the 4-part DNA nanostructure (blue for PBSII and red for Zif268). The split YFPs (nYFP and cYFP, grey) are fused to PSBII and Zif268 and co-expressed using pBAD on the pSC101 plasmid. When all parts are expressed, the proximity of the YFP domains forms the complete protein and fluorescence is detected. The split YFP (nYFP and cYFP) genetic sequences are added in Supplementary Table 4. (**b**) The impact of different combinations of r_oligo genes on the *in vivo* sensor are shown. The labels (1-part, 2-part and 3-part) refer to the genes present (r_oligo1, r_oligo1/r_oligo2 and r_oligo 1/r_oligo2/r_oligo3). In addition, the oligos were mutated to disrupt the operators (4-part_ZF_Mutant, four bases are mutated in the zif268 domain and five bases in the PBSII domain, coloured black in both domains). Finally, the system was expressed in the absence of HIVRT (4-part_-HIVRT). In all the experiments, 10 mM IPTG and 2 mM L-ara are added. Data shown represent the averages of three independent experiments performed on different days. The cytometry data and detailed schematic for each system are shown in Supplementary Fig. 10. (**c**) The increase in fluorescence over time is shown for the 4-part system when the sensor is expressed in the absence (-HIVRT) and presence of HIVRT (+HIVRT). The inducers (10 mM IPTG and 2 mM L-ara) are added at time 0 h. (**d**) Fluorescence of each different 4-part substructures (a–f) is compared with the fluorescence of the 4-part structure (b). The Δ*G* values are calculated using the model described in Supplementary Fig. 12 and based on values from NuPACK^70^. In all experiments, 10 mM IPTG and 2 mM L-ara are added. Data shown represent the averages of three independent experiments performed on different days. The cytometry data and detailed schematic for each system are shown in Supplementary Fig. 13. The ssDNA sequences are in Supplementary Table 2.

To assay intracellular assembly, we developed a genetically encoded sensor that detects the proper formation of the crossover motif in living cells. Two halves of split yellow fluorescence protein^60^ (nYFP and cYFP) linked to zinc-finger proteins (PBSII and Zif268) have been previously co-expressed and used as a sensor (Fig. 5a). Each of the r_oligo genes contains a zinc finger operator sequence and the expression of the four-part structure leads to their formation at a proximal distance from each other in the structure. For example, the Zif268 sequence is formed through the assembly of ssDNA1 with ssDNA4 (red), whereas the PBSII sequence is formed through the assembly of ssDNA2 with ssDNA3 (blue). This system is designed such that only the full formation of the desired four-part assembly leads to the reconstitution of the YFP. When all four ssDNAs are expressed, fluorescence is ninefold higher when HIVRT is present compared with when it is not (Fig. 5b). When HIVRT is expressed, but various ssDNAs are missing, no fluorescence is observed. Mutations made to the ZFP operator sequences also eliminate fluorescence. Finally, controls were performed to demonstrate that the operators can be placed on a plasmid to recruit the split YFP fragments, but the operators are too far apart on the ssDNA-containing plasmid to reform YFP (Supplementary Fig. 11).

The split-YFP system also enables the measurement of the assembly dynamics (Fig. 5c). Over a 6-h period, we observe that there is a continual increase in the fluorescence due to YFP reconstitution. This shift is indicative of the formation of the DNA nanostructure. As a control, we repeated this experiment in the absence of HIVRT and observed no increase in fluorescence over time.

Finally, we used the split-YFP system to confirm that all eight DNA crossover motifs are properly formed in the four-part nanostructure. This addresses the concern that partial structure formation (for example, where only six crossovers are formed) could facilitate the reconstitution of YFP. To determine if this occurs, we decomposed the four-part nanostructure into six substructures (shown as a–f in Fig. 5d) that consist of different numbers of crossover motifs. All of these structures retain the two operators (blue and red) that bind to the ZFPs that could lead to the reconstitution of YFP. However, because the substructures have differing stability, we hypothesized that this could affect the number of YFPs reconstituted. Indeed, we observe a correlation between the fluorescence and the calculated stability of the structure (Fig. 5d and Methods). The maximum fluorescence is observed for the complete structure that contains all eight crossover motifs.

## Discussion

This work demonstrates the ability to express multiple ssDNAs *in vivo* and their assembly into DNA nanostructures. Achieving this required two innovations. First, the optimization of the t-RNA^LYS^ hairpin so that it can be used as a genetic part (HTBS) that both serves as a transcriptional terminator and recruits HIVRT. Second, the co-expression of HIVRT and MLRT is important in enhancing ssDNA production. The genetic encoding of DNA nanostructures allows them to be produced on demand for *in vivo* applications. The ability to genetically encode imaging agents (fluorescent proteins)^62^ and optogenetic controls (phytochromes and rhodopsins)^63^ has revolutionized the ability to quantify and perturb cellular processes. Our platform provides a path to the construction of desired structures in cells. A number of creative *in vivo* applications have been shown for DNA nanostructures and ssDNA transformed into cells. For example, they have been used to sense intracellular conditions (for example, pH)^28^, super resolution imaging^64^ and the improvement of short interfering RNA effectiveness through their spatial organization^29^. In addition, genome editing methods based on oligo transformation, such as MAGE, require the co-transformation of multiple short ssDNAs^65^. This process could be accelerated by synthesizing single large constructs that contain many genetically encoded ssDNAs that are expressed *in vivo* for recombineering. Finally, metabolic pathways have been improved by scaffolding enzymes using proteins, RNA and plasmids in order to improve flux and avoid intermediate accumulation^60^,^66^,^67^. *In vivo* DNA nanotechnology could organize enzymes into arbitrary undegradable superstructures, perhaps to implement compartmentalization similar to zeolite catalysts or natural protein microreactors^68^.

This platform provides a path to making the complex products of DNA nanotechnology, including three-dimensional structures, DNA origami (through the co-expression of a single-stranded phage genome), nanomachines, and computation by strand displacement. It also serves as a good scaffold for the integration of protein and RNA elements in order to add sensing and functional capabilities to a composite material. Producing these materials in cells is an important first step towards commercialization as they can be produced as fermentation products, rather than requiring the large-scale chemical synthesis of many high-quality oligonucleotides.

## Methods

### Terminator strength experiments

Cells were inoculated in 200 μl LB Miller Broth in a 96-well plate covered with a breathable membrane (AeraSeal, Excel Scientific) and grown at 37 °C and 1,000 r.p.m. (Innova Shaker, Eppendorf) for 16 h. Overnight cultures were diluted 200-fold by mixing 1 μl culture into 199 μl of LB Miller Broth containing 10 mM L-arabinose and antibiotics. The cultures were then incubated at 37 °C and 1,000 r.p.m. After 3 h, a 15-μl aliquot of culture is prepared for flow cytometry by adding 185 μl of 1 × PBS containing 2 mg ml^−1^ kanamycin to stop translation. The terminator strength is calculated by comparing the expression of two fluorescent reporters, one placed before and one after the HTBS part, to the expression of the two identical fluorescent reporters lacking the HTBS part. Terminators will affect the fraction of transcripts of both reporters and their ratio can be used to calculate the terminator strength^48^ (TS):


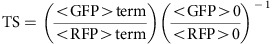


The subscript *Term* refers to the measurements when one of the HTBS sequences is present and 0 refers to the measurement of the control (pTS-Control). The control experiment is defined as a reporter plasmid lacking a terminator sequence between the two reporter genes. The plasmid maps are shown in Supplementary Fig. 14.

### Flow cytometry measurements

The assays were made using the LSR Fortessa (BD Biosciences) using the FITC (GFP) and PE-TxRed channels (RFP). The voltage gains for each detector were set to: FSC, 700 V; SSC, 241 V; FITC, 407 V; PE-TxRed, 650 V. Compensation was performed using cells that express only GFP or RFP. For each sample, at least 50,000 counts were recorded using a 0.5 μl s^−1^ flow rate. All data were exported in FCS3 format and processed using FlowJo (TreeStar Inc.). Data were gated by forward and side scatter. The fluorescence geometric mean of the gated population was calculated, and the mean auto-fluorescence of white cells was subtracted from the mean.

### Fluorescence assay for RT activity

Cells were inoculated in 500 μl LB Miller Broth with antibiotics in a 96-well plate covered with a breathable membrane (AeraSeal, Excel Scientific) at 37 °C at 1,000 r.p.m. (Innova Shaker, Eppendorf) for 16 h. Overnight cultures were diluted 200-fold by mixing 1 μl culture into 199 μl of LB medium containing 0.01 mM IPTG, 100 μg ml^−1^ spectinomycin and 100 μg ml^−1^ ampicillin. After 6 h of induction, a 10-μl aliquot of culture was prepared for cytometry by diluting it into 190 μl of 1 × PBS with 2 mg ml^−1^ kanamycin. The RFP ratio is calculated by dividing the fluorescence with the expression of the RT by that in the absence of the RT (cells containing the same plasmid, including inducible system, but lacking the HIVRT genes).

### Production and purification of DNA nanostructures

Cells were inoculated in 500 μl LB Miller Broth with antibiotics in a 96-well plate covered with a breathable membrane (AeraSeal, Excel Scientific) at 37 °C and 1,000 r.p.m. for 16 h. Overnight cultures were then diluted 1,000-fold by mixing 10 μl of the culture into 10 ml of LB Miller Broth containing the appropriate inducer and incubated at 37 °C and 250 r.p.m. for 18 h. After incubation, the DNA nanostructures were purified using the following protocol: (i) Cells were centrifuged at 5,000*g* for 7 min at 4 °C; (ii) the supernatant was removed; (iii) cells were resuspended in 200 μl of TE buffer (10 mM Tris-EDTA) containing 3 mg ml^−1^ of Lysozyme; (iv) 700 μl of RLT buffer (Qiagen, #79216) was added; (v) the resulting solution was centrifuged at 21,130 *g* for 2 min in order to remove the insoluble materials and the supernatant is transferred to a clean tube; (vi) 500 μl of 100% ethanol was added to the supernatant; (vii) 700 μl of the sample was transferred into a QIAquick Spin Column (Qiagen, #1018215) and centrifuged at 15,000*g* for 15 s; (viii) step vii was repeated until all the supernatant solution from step vi has passed through the same column tube (the flow through was discarded after each step); (ix) 700 μl of RW1 buffer (Qiagen, # 1053394) was added to the collection tube and centrifuged for 15 s at 15,000*g* (the flow through was discarded); (x) 500 μl RPE buffer (Qiagen, # 1018013) was pipetted into the column tube and centrifuged for 15 s at 15,000*g* (the flow through was discarded); (xi) 500 μl Buffer RPE (Mat. 1018013 Qiagen) was pipetted into the column tube and centrifuged for 2 min at 15,000*g* (the flow through was discarded); (xii) the empty column tube was centrifuged for 1 min at 15,000*g*; (xiii) the column was placed into a clean tube and 50 μl of water was added; (xiv) the resulting solution was then incubated with RNase A (100 μg ml^−1^, Qiagen) in the presence of 150 mM NaCl^69^ to recover the DNA–RNA chimera or without salt to recover just the ssDNA. The purified DNA solutions were then run on a 15% non-denaturing precast polyacrylamide gel (15% Mini-PROTEAN TBE Precast Gel #456-5053, Bio-Rad) in a Tris-borate-EDTA (TBE) buffer solution, which included Tris base (89 mM, pH=7.9), boric acid (89 mM) and EDTA (2 mM). The different samples were mixed with the loading dye and loaded in the wells of the gel. The gels were run on Mini-PROTEAN Tetra Cell (Bio-Rad, #165-8000) under a constant voltage (100 V). After electrophoresis, the gel was stained with SYBR Gold nucleic acid gel stain (Invitrogen) and imaged. The band at the correct size was excised and page purified. The ladder used is 100 bp (New England BioLabs, Mat. N3231L). The gel images were analysed using ImageJ. All the images have been inverted. The bands have been selected and converted to plot (plots lane function). The different surfaces under the plots (representing the assemblies) have been selected and converted to intensity using the wand-tracing tool. The gel background intensity was calculated and subtracted from the intensity values presented. This protocol has been used for experiments shown in Fig. 2b–d, Fig. 4b and Supplementary Figs 1,2a,4,7 and 9.

### DNase assay

The identical purified *in vivo* ssDNAs/DNA nanostructures (from step xiv of the ‘Production and purification of DNA nanostructures' experimental paragraph) were incubated with DNAse I (4 U, New England BioLabs, Mat. M0303S) at 25 °C for 4 h. Then, the samples were mixed with the loading dye and loaded in the wells of the gel. The resulting solutions were then run on a 15% non-denaturing precast polyacrylamide gel (15% Mini-PROTEAN TBE Precast Gel #456-5053, BI0-RAD) in a TBE buffer solution, which included Tris base (89 mM, pH=7.9), boric acid (89 mM) and EDTA (2 mM).

### Scale up of the *in vivo* ssDNA production

Cells were inoculated in 1 l of Terrific Broth containing the appropriate plasmids and incubated at 37 °C at 250 r.p.m. for 24 h. After incubation, the ssDNAs were purified using TRIzol Reagent protocol (Life Technology, # 10296010). RNAse A (100 μg ml^−1^, Qiagen) was then added to the resulting solutions and incubated at 37 °C for overnight. After RNA degradation, the solutions were cleaned and concentrated using oligo clean and concentrator kit (ZYMO Research Corp., D4061 ZYMO). This protocol has been used for experiments shown in Figs 2e, 3 and Supplementary Fig. 3. For a detailed *in vivo* 72-nt ssDNA production protocol, see Supplementary Methods.

### DNA self-assembly in solution

The *in vivo* purified ssDNAs (1 μM for the 1D chains or 2 μM for the 2D arrays) were dilutated with TAE-Mg^2+^ consisted of Tris (40 mM, pH 8.0), acetic acid (20 mM), EDTA (2 mM) and magnesium acetate (12.5 mM) buffer and slowly cooled from 95 to 22 °C over 48 h.

### DNA self-assembly on a surface

2.0 μM of the 2D arrays *in vivo* purified ssDNAs were dissolved in TAE-Mg2+ buffer and incubated at 95 °C for 5 min, at 65 °C for 1 h, at 50 °C for 1 h, at 37 °C for 1 h, at 22 °C for 1 h, at 32 °C for 1 h (Z-shaped tile). Five microlitres of annealed solution was transferred onto a preheated mica surface at 32 °C and incubated at 32 °C in a humidity chamber for 16 h.

### PAGE purification

The excised gel slice was incubated in 400 μl RNA Recovery Buffer (ZYMO Research Corp., R1070-1-10) at 65 °C for 15 min. The resulting solution was then placed into a Zymo-Spin IV Column (ZYMO Research Corp., C1007-50) and centrifuged at 9,391*g* for 30 s. The flow-through was then transferred into a tube including a 5 × volume of buffer PB (Qiagen) and 700 μl of the resulting solution is transferred into QIAquick Spin Columns (Qiagen, Mat. No. 1018215) and centrifuged at 9,391*g* for 30 s. This was repeated until all of the solution passed through the column and the flow through was discarded. Then, 750 μl of PE buffer (Qiagen) was added to the column and centrifuged at 9,391*g* for 30 s, the flow through discarded, and the empty column centrifuged at 9,391*g* for 1 min to remove residual PE buffer. The column was placed in a clean tube and the DNA is eluted by adding 50 μl of EB buffer (10 mM Tris-Cl, pH 8.5) followed by centrifugation at 9,391*g* for 1 min.

### Determination of the *in vivo* ssDNA titre

The presented titre is the total amount of ssDNA that we can purify from a culture of defined volume and expression time. A 1-l culture is grown for 24 h and the ssDNA is purified as described above. The purified ssDNA is then run on a denatured PAGE gel and compared with its identical chemically synthesized ssDNA (ordered from IDT). The appropriate band is then cut and purified from the gel. The DNA concentration is measured by measuring the absorbance (Abs, OD 260) using a ND-1000 Spectrophotometer Nanodrop of the resulting purified ssDNA. The absorbance value is converted to nanogram per microlitre using the Nanodrop software. The culture volume then divides the total weight of the ssDNA. To determine the total amount of material lost (MA) during purification, a control experiment was run by using a synthesized 49 nt oligo (3.2 μg; ordered from IDT) of known quantity that was then purified. The titre is calculated as (Abs (ng μl^−1^) × Volume of the PAGE purified ssDNA sample (μl)) × (Volume of the total *in vivo* stock divided by the Volume of the solution run on the PAGE) divided by the Volume of the cell culture (l)) × Constant (Material lost during the PAGE recovery). For our system, titre=(1.2 ng μl^−1^ × 10 μl) × (35 μl/1 μl)/1 l) × 10=4.2 μg l^−1^.

### Sequencing and deep sequencing experiments

A 10-ml culture was grown at 37 °C and 1,000 r.p.m. for 18 h and the ssDNA was purified as described above. The purified ssDNA was then run on a denatured PAGE gel and the appropriate band is cut and purified. The resulting purified ssDNA was then amplified using PCR with a high fidelity polymerase (Qiagen) for 33 PCR cycles, involving denaturation for 15 s at 95 °C, annealing for 30 s at 72 °C and primer extension for 30 s at 65 °C. The solution was then run on a 1% agarose gel and the appropriate band purified. The resulting solution was then sequenced (Quintarabio) and deep sequenced via deep sequencing of PCR amplicons methodology (DNA Core Facility, MGH). The error rate of the RTs was calculated as the number of mutagenesis errors divided by the total number of bases: Error rate=57,785/67,427,575=8.56 × 10^−4^.

### Short *in vivo* ssDNA sequencing using DNA adapters

The short 72-nt *in vivo* produced ssDNA sequencing experiment using the DNA adapters methodology^53^ was performed by using the following protocol: Step 1—Dephosphorylation and heat denaturation. Add 1 μl of FastAP (1 U) to the 42-μl reaction mixture (20 μl of water, 8 μl of CircLigase buffer II (10 × ), 4 μl of MnCl_2_ (50 mM) and 10 μl of the 72-nt PAGE purified *in vivo* ssDNA). Incubate the reaction in a thermal cycler with a heated lid for 10 min at 37 °C, and then at 95 °C for 2 min. Quickly transfer the tubes into an ice-water bath for 1 min. Step 2—Ligation of the first adapter. Add 32 μl of PEG-4000 (50%), 1 μl of Adapter oligo CL78 (10 μM) and 4 μl CircLigase II (100 U μl^−1^) to the resulting solution from step 1 to obtain a final reaction volume of 80 μl. Then, incubate the reaction mixtures in a thermal cycler with a heated lid for 1 h at 60 °C, and then add 2 μl of stop solution (98 μl of 0.5 M EDTA (pH=8.0) and 2 μl of Tween-20). Step 3—Immobilization of ligation products on beads. First, prepare the beads by transferring 20 μl from the bead stock solution (MyOne C1) into a 1.5-ml tube. Pellet the beads using a magnetic rack, discard the supernatant and wash the beads twice with 500 μl of bead-binding buffer (7.63 ml of water, 2 ml of 5 M NaCl, 100 μl of 1 M Tris-HCl (pH 8.0), 20 μl of 0.5 M EDTA (pH 8.0), 5 μl of Tween-20 and 250 μl of 20% (wt/vol) SDS), and then resuspend the beads in 250 μl of bead-binding buffer. In parallel, incubate the ligation reaction from step 2 for 1 min at 95 °C in a thermal cycler with a heated lid, and then transfer the tube into an ice-water bath for 1 min. Finally, transfer the ligation reaction to the bead solution and rotate the tube for 20 min at room temperature. Step 4—Primer annealing and extension. Pellet the beads from step 3 using a magnetic rack and discard the supernatant. Wash the beads once with 200 μl of wash buffer A (47.125 ml of water, 1 ml of 5 M NaCl, 500 μl of 1 M Tris-HCl (pH 8.0), 100 μl of 0.5 M EDTA (pH 8.0), 25 μl of Tween-20 and 1.25 ml of 20% (wt/vol) SDS) and once with 200 μl of wash buffer B (8.375 ml of water, 1 ml of 5 M NaCl, 500 μl of 1 M Tris-HCl (pH 8.0), 100 μl of 0.5 M EDTA (pH 8.0) and 25 μl of Tween-20). Then, resuspend the beads with 47 μl of the reaction mixture (40 μl of water, 5 μl of isothermal amplification buffer (10 × ), 0.5 μl of dNTP mix and 1 μl of the extension primer CL9 (100 μM)). Incubate the tube in a thermal shaker for 2 min at 65 °C and place the tube in an ice-water bath for 1 min. Then, immediately transfer the tube to a thermal cycler precooled to 15 °C followed by the addition of 3 μl of Bst 2.0 polymerase (24 U). Incubate the reaction mixtures by increasing the temperature by 1 °C per min, ramping it up from 15 °C to 37 °C, while implementing a final incubation step of 5 min at 37 °C. Step 5—Blunt-end repair. Pellet the beads from step 4 using a magnetic rack and discard the supernatant. Wash the beads once with 200 μl of wash buffer A. Resuspend the beads in 100 μl of stringency wash buffer (9.5 ml of water, 250 μl of 20% (wt/vol) SDS and 250 μl of 20 × SSC buffer) and incubate the bead suspensions for 3 min at 45 °C in a thermal shaker. Pellet the beads using a magnetic rack and discard the supernatant. Wash the beads once with 200 μl of wash buffer B. Pellet the beads using a magnetic rack and discard the wash buffer. Add 99 μl of the reaction mixture (86.1 μl of water, 10 μl of Buffer Tango (10 × ), 2.5 μl l of Tween-20 (1%) and 0.4 μl of dNTP mix) to the pelleted beads and resuspend the beads by vortexing. Add 1 μl of T4 DNA polymerase (5 U). Finally, incubate the reaction mixtures for 15 min at 25 °C in a thermal shaker and stop the reaction by adding 10 μl of EDTA (0.5 M). Step 6—Ligation of second adapter and library elution. Pellet the beads using a magnetic rack and discard the supernatant. Wash the beads with 200 μl of wash buffer A, 200 μl of stringency wash buffer (with incubation at 45 °C for 3 min) and 200 μl of wash buffer B. Pellet the beads using a magnetic rack and discard the supernatant. Add 98 μl of the reaction mixture (73.5 μl of water, 10 μl of T4 DNA ligase buffer (10 × ), 10 μl of PEG 4000(50%), 2.5 μl of Tween-20 (1%) and 2 μl of the double-stranded adapters (100 μM of pre-hybridized oligos CL53 and CL73)). Mix and add 2 μl of T4 DNA ligase. Finally, incubate the solution for 1 h at room temperature. Step 7—De-immobilization of the DNA from the beads. Pellet the beads from step 6 using a magnetic rack and discard the supernatant. Wash the beads once with 200 μl of wash buffer A. Resuspend the beads in 100 μl of stringency wash buffer and incubate the bead suspensions for 3 min at 45 °C in a thermal shaker. Pellet the beads using a magnetic rack and discard the supernatant. Wash the beads once with 200 μl of wash buffer B. Pellet the beads using a magnetic rack and discard the wash buffer. Add 25 μl of TET buffer (49.375 ml of water, 500 μl of 1 M Tris-HCl, 100 μl of 0.5 M EDTA and 25 μl of Tween-20) to the pelleted beads, resuspend the beads by vortexing. Incubate the bead suspensions for 1 min at 95 °C in a thermal cycler with a heated lid. Immediately transfer the tube to a magnetic rack. Finally, transfer the supernatant, which contains the 72-nt *in vivo* produced ssDNA conjugated to the DNA adapters, to a fresh tube. Step 8—PCR amplification and sequencing. PCR the 72-nt *in vivo* produced ssDNA conjugated to the DNA adapters by adding 12 μl of the 72-nt *in vivo* produced ssDNA conjugated to the DNA adapters (resulting solution from step 7), 28.5 μl of water, 5 μl of AccuPrim Pfx reaction mix (10X), 2 μl of P7L primer, 2 μl of P5 primer and 1 μl of AccuPrime Pfx polymerase (2.5 U μl^−1^). Incubate the reactions in a thermal cycler with the following thermal profile. Initial denaturation should be carried out at 95 °C for 2 min. Follow this by 27 of PCR cycles, involving denaturation for 15 s at 95 °C, annealing for 30 s at 60 °C and primer extension for 1 min at 68 °C. Finally, terminated the PCR reaction by incubating the solution at 72 °C for 5 min. The resulting PCR solution was then sequenced (Quintarabio) with primer SEQ. Note that oligo P7L was extended in its middle with additional 35-nt (5′- TTGTTTTTCTTTGTTTCTTTTTCTTGTCTTTCTTT -3′. See Supplementary Table 5 for the complete P7L oligo sequence and all the other oligos used for these assays. This extension had been added to increase the size of the product, thus, allowing the *in vivo* ssDNA to be fully sequenced. The second oligo used in this assay (P5) was changed to be partially complementary to the *in vivo* ssDNA and the DNA adapter. A control experiment had been performed in parallel in which the *in vivo* ssDNA was replaced with the same sequence synthesized by IDT (commercial).

### AFM assays

AFM measurements were performed at room temperature using Dimension 3100 D31005-1 with Nanoscope V (Veeco). AFM images were recorded on freshly cleaved mica surfaces (TED PELLA, Inc.). A 10-μl aliquot of the solution containing the DNA nanostructures was deposited in the presence of 10 mM Mg(Ac)_2_. The surfaces were rinsed with 10 mM Mg(Ac)_2_ solution and dried under a stream of air. Images were recorded with AFM tips (Model NSC11, Umasch, and Model RTESP, Part MPP-11100-10, BRUKER) and using tapping mode at their resonance frequency. The images were analysed using NANO Scope analysing software (Vecco). The nanostructures chosen for evaluations were auto-selected and analysed using NANO Scope analysing software (Vecco). More specifically, all particles with a minimum size of 0.5 nm and a maximum size of 3 nm were auto-selected from the AFM images.

### Fluorescence assay for the split GFP assay

Cells were inoculated in 500 μl LB Miller Broth with antibiotics in a 96-well plate covered with a breathable membrane (AeraSeal, Excel Scientific) at 37 °C at 1,000 r.p.m. (Innova Shaker, Eppendorf) for 16 h. Overnight cultures are diluted 200-fold by mixing 2 μl culture into 198 μl of LB medium containing 10 mM IPTG, 2 mM L-ara, 50 μg ml^−1^ spectinomycin, 25 μg ml^−1^ kanamycin and 50 μg ml^−1^ ampicillin. After 10 h of induction, a 3-μl aliquot of culture is prepared for cytometry by diluting it into 197 μl of 1 × PBS with 2 mg ml^−1^ kanamycin.

### Materials and DNA sequences

Materials, plasmid maps and DNA sequences are provided in the Supplementary Methods section, Supplementary Figs 14–22 and Supplementary Tables 1–4.

## Additional information

**Accession codes:** Deep-sequencing data of the reverse transcriptase products have been deposited at the Sequence Read Archive under the accession code SRP070443.

**How to cite this article:** Elbaz, J. *et al*. Genetic encoding of DNA nanostructures and their self-assembly in living bacteria. *Nat. Commun.* 7:11179 doi: 10.1038/ncomms11179 (2016).

## Supplementary Material

Supplementary Information Supplementary Figures 1-22, Supplementary Tables 1-5, Supplementary Methods and Supplementary References
