# Supplementary material for: Genetic encoding of DNA nanostructures and their self-assembly in living bacteria
Source: Nat Commun. 2016 Apr 19;7:11179. doi: 10.1038/ncomms11179 (PMC4838831; doi:10.1038/ncomms11179)
Supplement: Supplementary Information — Supplementary Figures 1-22, Supplementary Tables 1-5, Supplementary Methods and Supplementary References [file ncomms11179-s1.pdf]

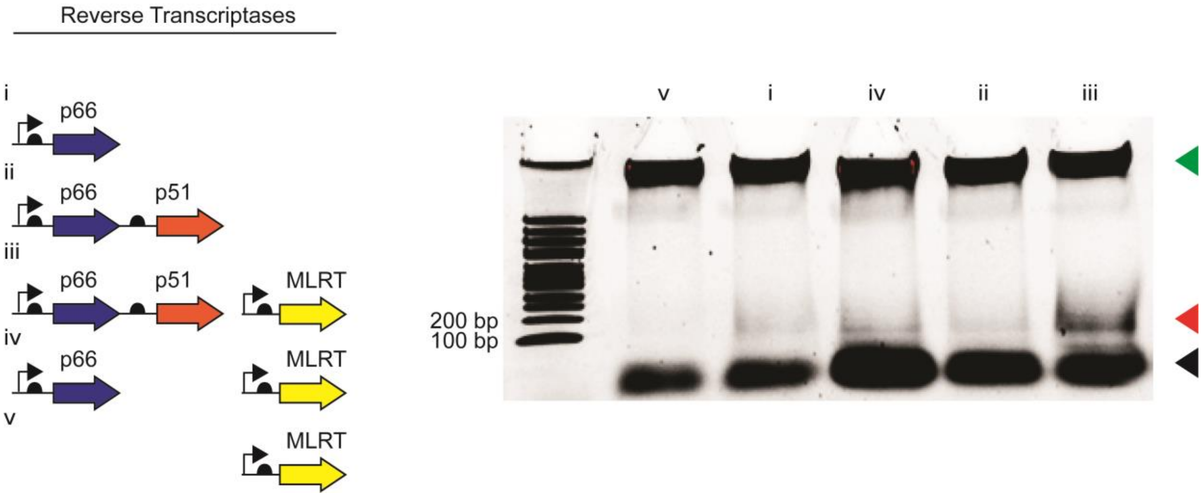


**Supplementary Figure 1: The impact of different combinations of RT expression on ssDNA production.** The full gel used to make Figure 2b is shown. The numbers above the gels correspond to the combination of RTs tested (left). The red triangle shows the predicted location of the ssDNA. The green triangle shows the predicted location of the plasmid. The black triangle shows the location of degraded RNAs.

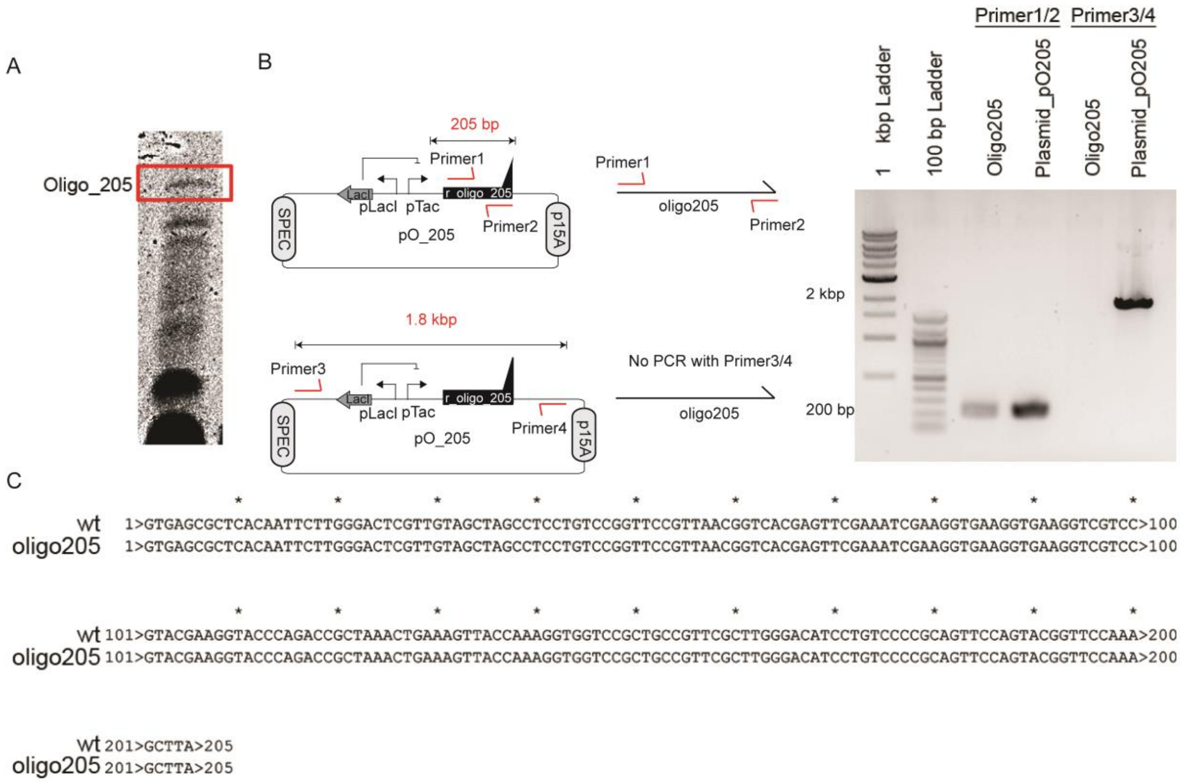


**Supplementary Figure 2: Production, purification, and sequencing of a 205 nt oligo. (A)** Denatured PAGE of the oligo_205 purified from cell culture under conditions removing the HTBS (RNase, no salt). The red band represents the band cut, purified, PCR, and sequenced. (**B**) The purified oligo_205 was recovered by PCR using primers 1 and 2 (red, PCR I) and run in a 1% agarose gel. A band around 200 bp was observed, confirming the correct size of the *in vivo*-produced oligo. As a control experiment, another PCR reaction was run with primers 1 and 2 while exchanging oligo205 with the plasmid pO_205 (this plasmid includes the identical DNA sequence as oligo_205). An identical PCR product was generated and visualized in the 1% agarose gel. An additional control experiment was run while both the oligo_205 and plasmid pO_205 were PCR with primers 3 and 4 (PCR II). These reactions should enable a 1.8 kbp PCR product only in the presence of pO205 plasmid. As expected, no PCR product was detected using oligo205 as the template, confirming that the purified oligo_205 sample does not contain plasmid contamination. (**C**) Sequencing analysis of the oligo_205 compared to its predicted sequence.

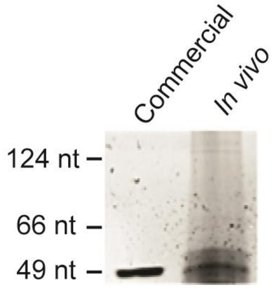


**Supplementary Figure 3: Characterization of an *in vivo* produced 49 nt oligo.** Denatured PAGE of a 49-nt *in vivo* ssDNA product compared with industrial chemically synthesized ssDNA is shown. The ladder was calculated using commercial oligos run in the same gel. The 49-nt *in vivo* ssDNA was further used for the assembly of the 2D arrays shown in Figure 3.

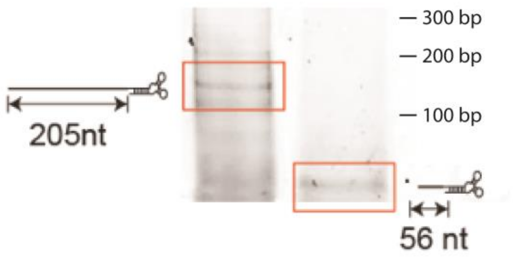


**Supplementary Figure 4: Prodution of *in vivo* ssDNAs of different sizes.** Non-denatured PAGE of a 205 nt ssDNA and a 56 nt ssDNA preserving the HTBS is shown.

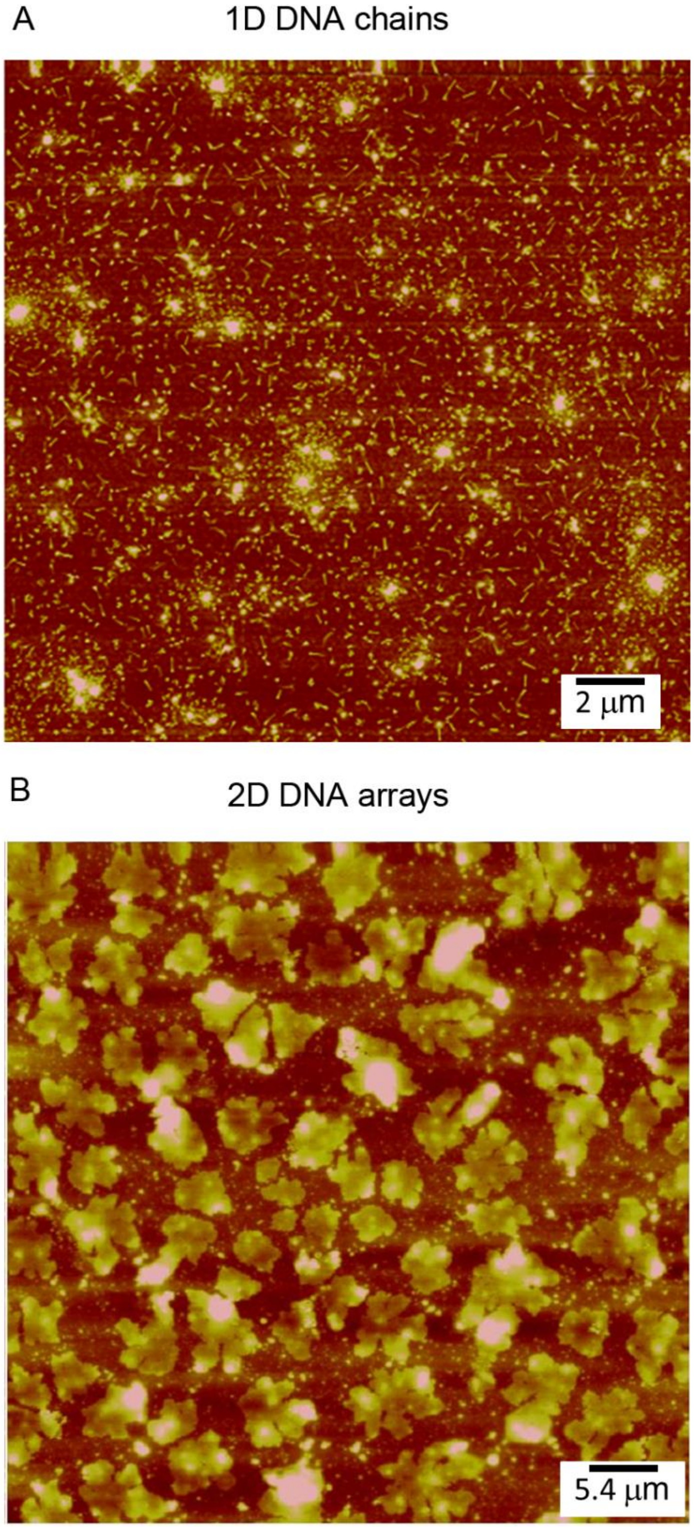


**Supplementary Figure 5: Large AFM images of the 1D DNA chains and 2D DNA arrays.** (**A**) AFM image of the 1D chains using the solution assembly method is shown. (**B**) Large scale AFM image of the 2D arrays using the solution assembly method is shown (Methods).

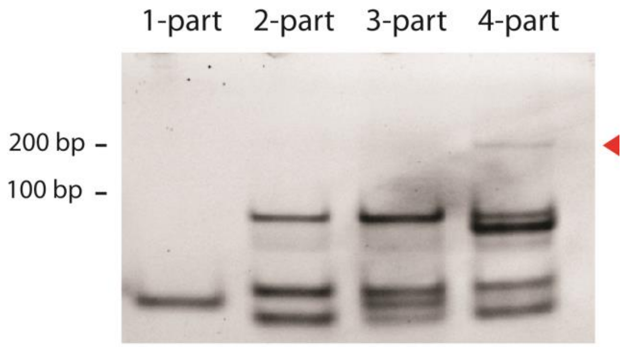


**Supplementary Figure 6:** ***in vitro*** **DNA crossover nanostructure assembly using chemically synthesized ssDNAs.** Assembly of the DNA nanostructures is shown for different combinations of synthetic chemically synthesized DNAs (from IDT in HPLC purified form) assembled *in vitro* at 37°C for 18 h (Correspond to the temperature and time of in vivo assembly): (1-part) DNA sequence of r_oligo1; (2-part) DNA sequences of r_oligo2 and r_oligo3; (3-part) DNA sequences of r_oligo1, r_oligo2 and r_oligo3; (4-part) DNA sequences of r_oligo1, r_oligo2, r_oligo3. And r_oligo4. A unique band is observed in the presence of all four oligos forming the DNA crossover nanostructure (red triangle). Note that similar intermediate assembly phenomena were observed with *in vivo* DNA nanostructure production (Figure 4).

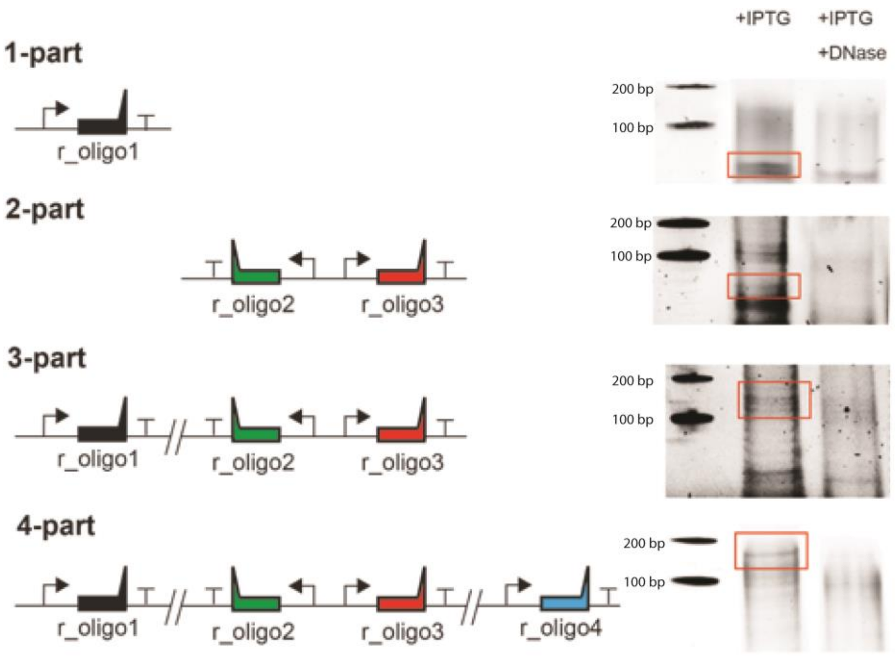


**Supplementary Figure 7: DNase control experiments with the 4-part assembly.** In Figure 4b, the +-IPTG experiments were performed on different days from the DNAse control (experiments shown here). This figure shows the full gels before and after the addition of DNAse. The production of the DNA nanostructures are shown when different combinations of r_oligo genes are expressed in the presence of IPTG (+IPTG, 1 mM) under purifications conditions preserving the HTBS.

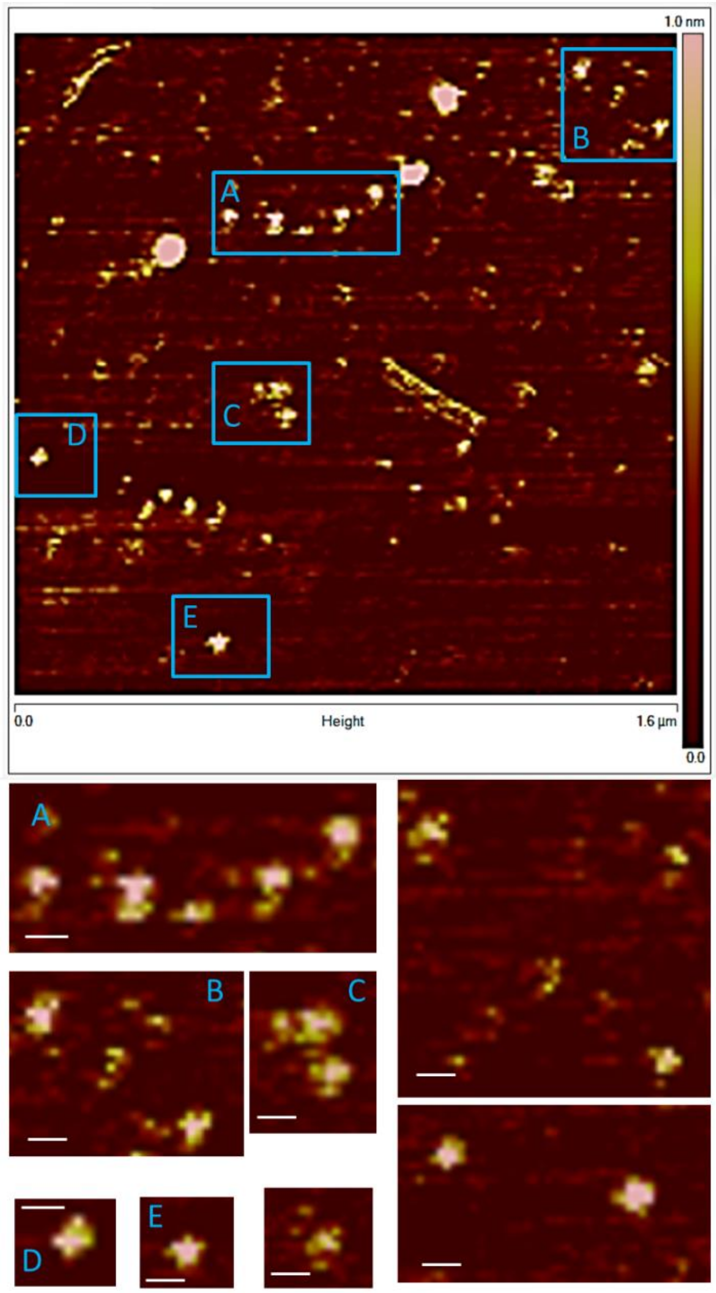


**Supplementary Figure 8: Detailed AFM images of the 4-part DNA nanostructures.** These images and those used to make Figure 4 were performed on different days from different starting cultures. The top panel shows large scale AFM image (1.6 µm). The bottom magnifies zoom into specific structures (scale bar equal 50 nm). Letters correspond to regions within the larger image (no letter indicates that the detailed images were recorded from other AFM images). The experiments were performed with AFM tips (Model NSC11, Umasch, USA) and using tapping mode at their resonance frequency. The images were analyzed using NANO Scope analyzing software (Vecco, USA).

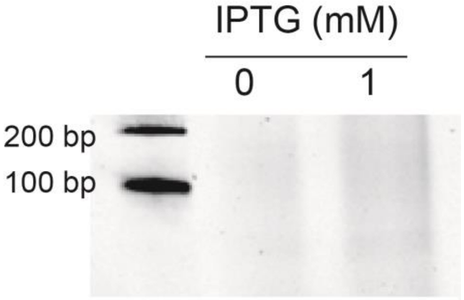


**Supplementary Figure 9: HIVRT control experiment for the 4-part DNA nanostructure.** Data for the 4-part system lacking the HIVRT gene were generated under same condition to the 4-part system expressing the DNA nanostructure shown in Figure 4. As expected, no bands are detected and no ssDNA are expressed in the absence of the HIVRT gene.

**Supplementary Figure 10 (next page): Expanded schematics and cytometry data corresponding to Figure 5b.** The 4-part DNA nanostructure includes eight 10-bp binding domains that connect the four ssDNA strands (ssDNA1−ssDNA4). One of the 10-bp domain forms the Zif268 zinc finger sequence through the hybridization of ssDNA1 with ssDNA4 (9 bp, red). Another 10-bp domain forms the PBSII zinc finger sequence through the hybridization of ssDNA2 with ssDNA3 (9 bp, blue). To complete a single turn helix, a single base pair (black) was added to both of these domains. In parallel to the expression of the different ssDNAs, two split-YFP proteins (nYFP and cYFP) linked to zinc finger proteins (nYFP with PBSII and cYFP with Zif268) were co-expressed. Controls were performed, including the expression of the 4-part system in the absence of HIVRT and those lacking different ssDNAs. Finally, 4-bp mutations to zif268 and 5-bp mutations to PBSII (colored black in the 4-part_ZF_Mutant structure) were added to the two zinc finger domains, and this eliminated fluorescense. The histogram of the 4-part system lacking the split-YFP genes (4-part_-sliptYFPs) is also shown. The ssDNA sequences are provided in Supplementary Table 2.

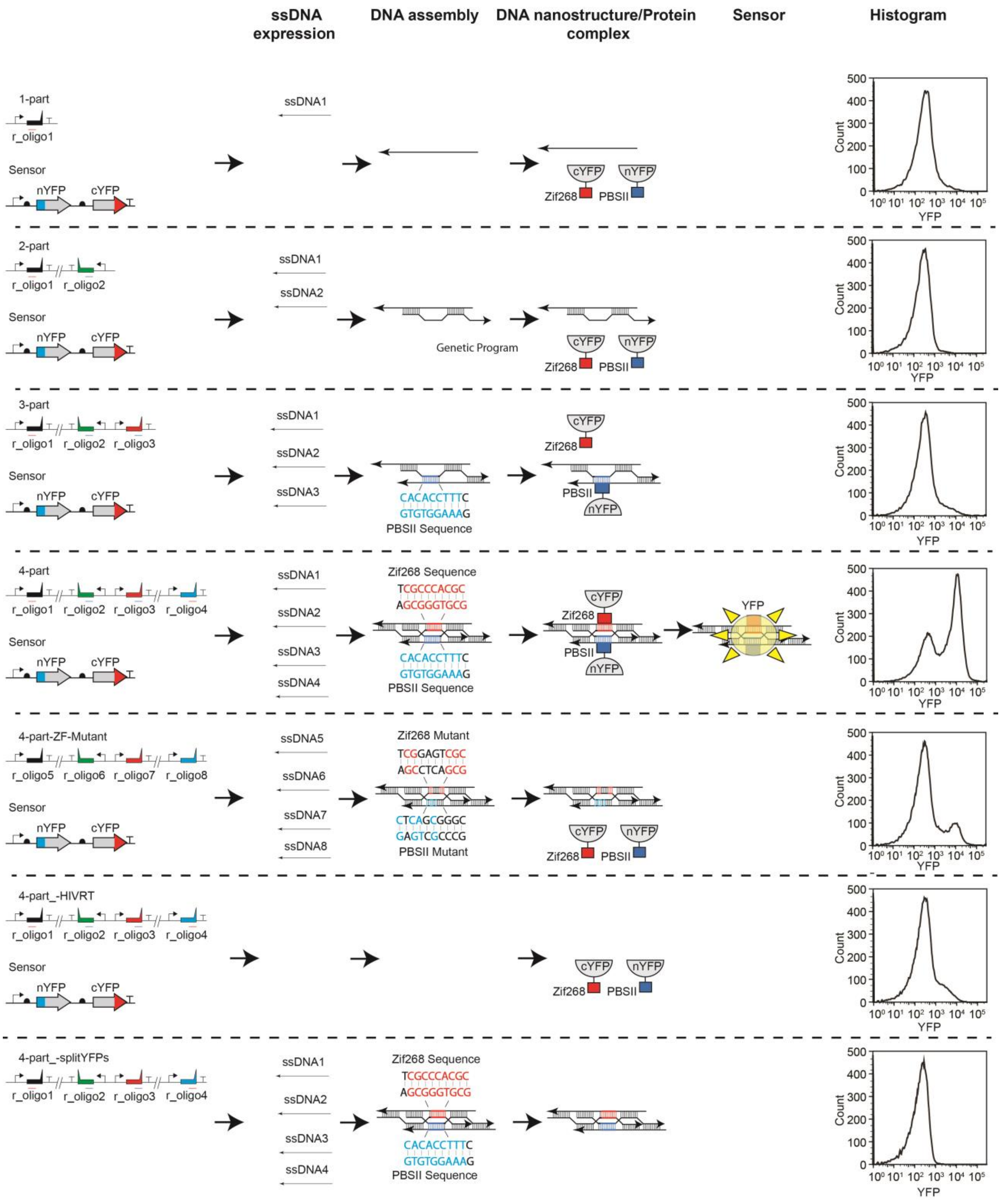
ssDNA expression
DNA assembly
DNA nanostructure/Protein complex
Sensor
Histogram
1-part
r_oligo1
Sensor
nYFP
cYFP
ssDNA1
cYFP
nYFP
Zif268
PBSII
2-part
r_oligo2
ssDNA2
Genetic Program
3-part
r_oligo3
ssDNA3
CACACCTTTC
GTGTGGAAAG
PBSII Sequence
4-part
r_oligo4
ssDNA4
Zif268 Sequence
TCGCCCACGC
AGCGGGTGCG
YFP
4-part-ZF-Mutant
r_oligo5
r_oligo6
r_oligo7
r_oligo8
ssDNA5
ssDNA6
ssDNA7
ssDNA8
Zif268 Mutant
TCGGAGTCGC
AGCCTCAGCG
CTCAGCGGGC
GAGTCGCCCG
PBSII Mutant
4-part_-HIVRT
4-part_-splitYFPs
Count
YFP

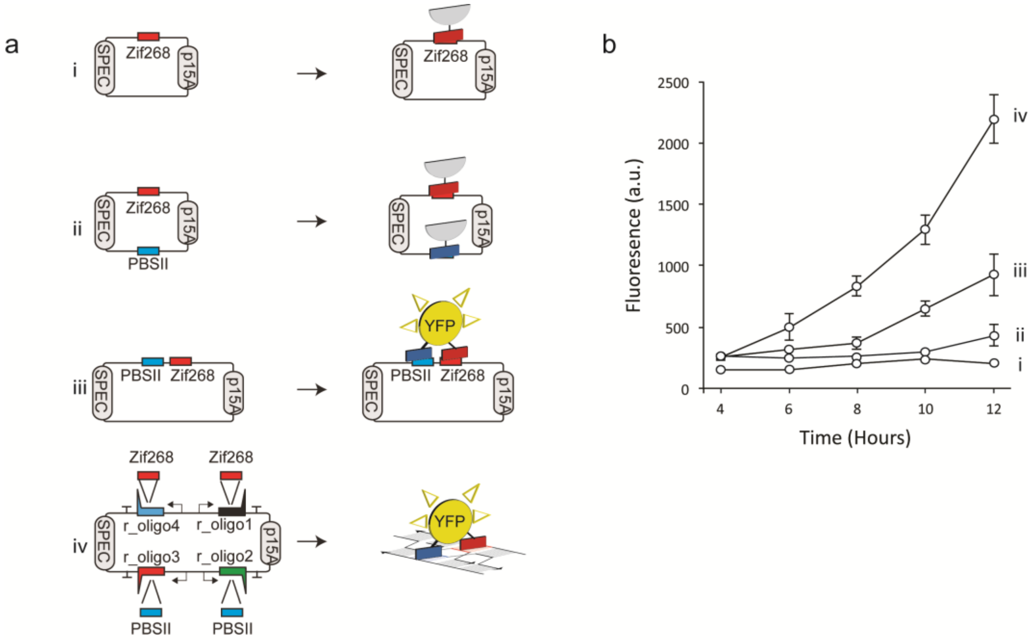


**Supplementary Figure 11: Control experiment of the the 4-part system compared with a linear plasmid used to reconstitute YFP.** (**a**) Schematic for the different systems investigated to confirm that reconstitution of the YFP is only due to the 4-part assembly and not to a linear plasmid that includes the ZF sequences[3]. Each system includes a plasmid with a different combination of the ZF domains and at different distances from each other: : i) a single ZF domain, ii) two ZF domains at a long distance from each other (1426 and 2007 bp), iii) the two ZF domain at a close distance from each other (2bp), iv) the 4-part oligo plasmid that includes the two ZF domains at a long distance from each other (1426 and 2007 bp). As previously demonstrated[4], a linear plasmid that includes the two ZF domains at a short distance (2 bp) may be used to reconstitute YFP (iii). Thus, the 4-part oligo plasmid has been designed in such a way that the two different ZF domains are located at a long distance from each other (1426 and 2007 bp), thus preventing the reconstitution of YFP (iv). (**b**) Increase in fluorescence over time is shown for the different systems shown in a. These data demonstrate that the plasmids with one or more ZF domains at a long distance do not reconstitute YFP (i+ii). In addition, the production of the 4-part DNA nanostructure (iv) reconstitutes 2-3 times more YFP compared with a linear plasmid that includes the ZF domains in close proximity (iii). Data shown represent the averages of three independent experiments performed on different days. Note that all the experiments were performed in the presence of the HIVRT plasmid (pHIV_pTp66p51) and the MLRT-split YFPs plasmid (pMLRT_sYFP) to maintain the identical 4-part DNA nanostructure *in vivo* sensor conditions (Methods).

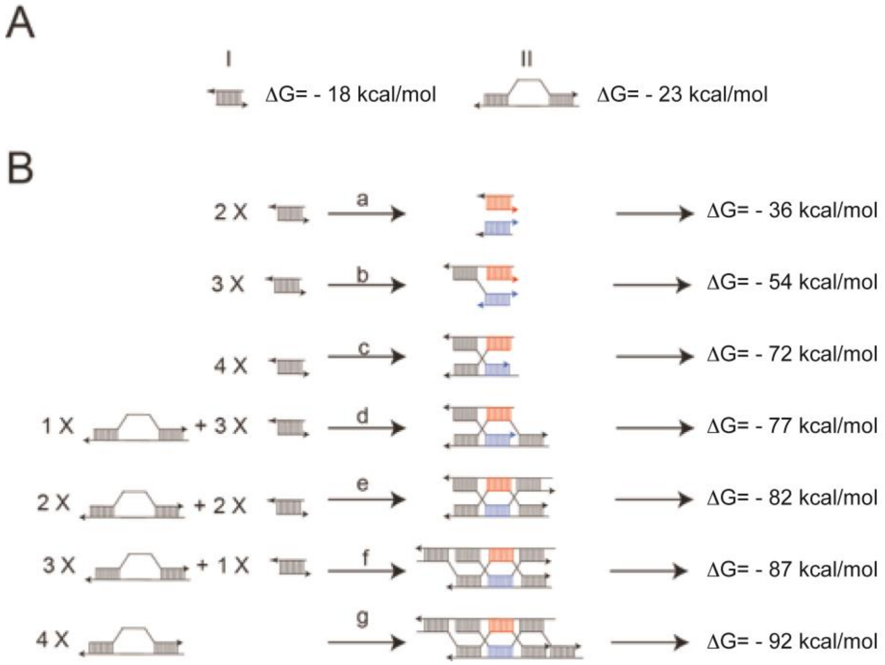


**Supplementary Figure 12: Free enregy model for the different structures shown in Figure 5d.** (**A**) The free energies of a 10-bp motif (I, −18 kcal/mol) and a two 10-bp motif separated by a 10-base loop (II, −23 kcal/mol) are shown. The free energy has been calculated using Nupack[4]. (**B**) Each of the different DNA assemblies (Figure 5d) has been decomposed according to the number of motifs shown in A. Then, the appropriate free energy of each substructure was calculated by adding the number of motifs included in the different structures. For example, assembly a is formed by two "I" motifs ($\Delta G = 2x(-18) = -36$ kcal/mol) and assembly (f) is formed by one "I" and three "II" motifs ($\Delta G = 1x(-18) + 3x(-23) = -87$ kcal/mol).

**Supplementary Figure 13 (next page): Detailed description of the data shown in Figure 5d.** Six different 4-part oligo plasmids have been constructed and have been used to express the different 4-part substructures (4-part-a, 4-part-b, 4-part-c, 4-part-d, 4-part-e, and 4-part-f). For the sequences of the different r_oligo genes, see Supplementary Table 2, and for their plasmid maps, see Supplementary Fig. 22.

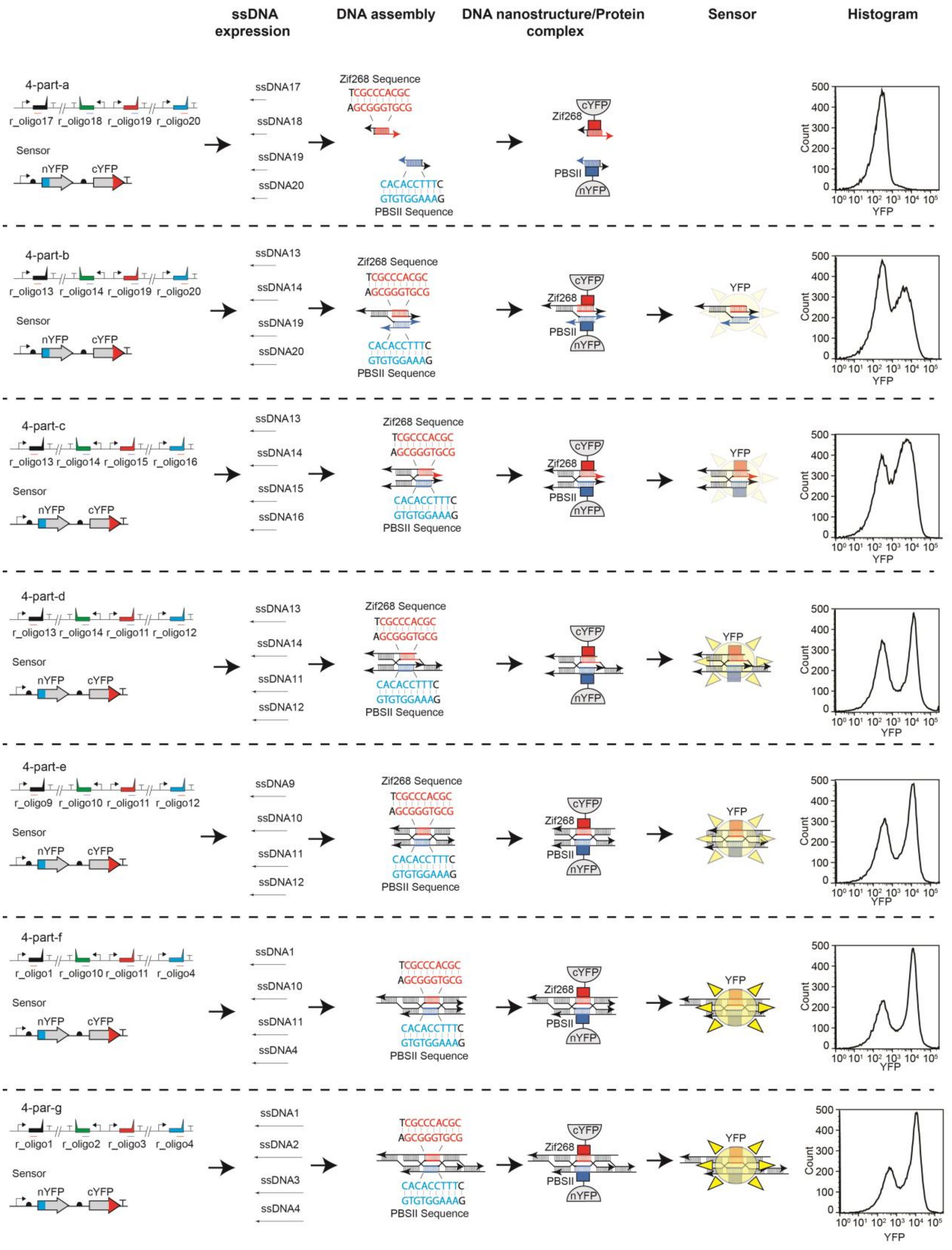
ssDNA expression
DNA assembly
DNA nanostructure/Protein complex
Sensor
Histogram
4-part-a
r_oligo17 r_oligo18 r_oligo19 r_oligo20
Sensor
nYFP
cYFP
ssDNA17
ssDNA18
ssDNA19
ssDNA20
Zif268 Sequence
TCGCCCACGC
AGCGGGTGCG
CACACCTTTC
GTGTGGAAAG
PBSII Sequence
Zif268
PBSII
4-part-b
r_oligo13 r_oligo14 r_oligo19 r_oligo20
ssDNA13
ssDNA14
ssDNA19
ssDNA20
YFP
4-part-c
r_oligo13 r_oligo14 r_oligo15 r_oligo16
ssDNA13
ssDNA14
ssDNA15
ssDNA16
4-part-d
r_oligo13 r_oligo14 r_oligo11 r_oligo12
ssDNA13
ssDNA14
ssDNA11
ssDNA12
4-part-e
r_oligo9 r_oligo10 r_oligo11 r_oligo12
ssDNA9
ssDNA10
ssDNA11
ssDNA12
4-part-f
r_oligo1 r_oligo10 r_oligo11 r_oligo4
ssDNA1
ssDNA10
ssDNA11
ssDNA4
4-par-g
r_oligo1 r_oligo2 r_oligo3 r_oligo4
ssDNA1
ssDNA2
ssDNA3
ssDNA4
Count
YFP

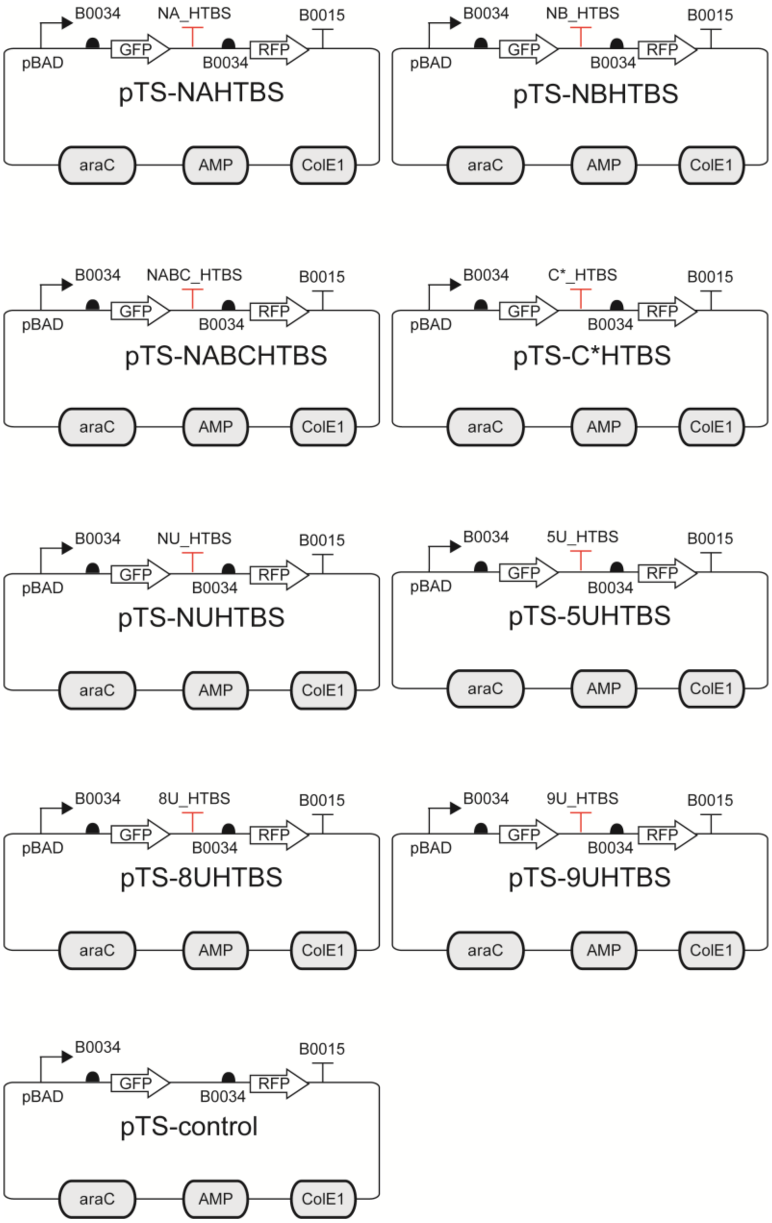


**Supplementary Figure 14: HTBS terminator strength (Ts) measurement plasmids maps**. These were used for the experiments shown in Figure 1c. Part sequences are provided in Supplementary Tables 1-4. Parts beginning with “B” are from the Registry of Standard Biological Parts. The HTBS variants are named as in Supplementary Table 1. The plasmid construct includes an arabinose-inducible PBAD promoter placed upstream of two genes, the green fluorescent protein gene (*gfp*) and the gene encoding red fluorescent protein (*rfp*). The different HTBS sequences were placed in between the two genes.

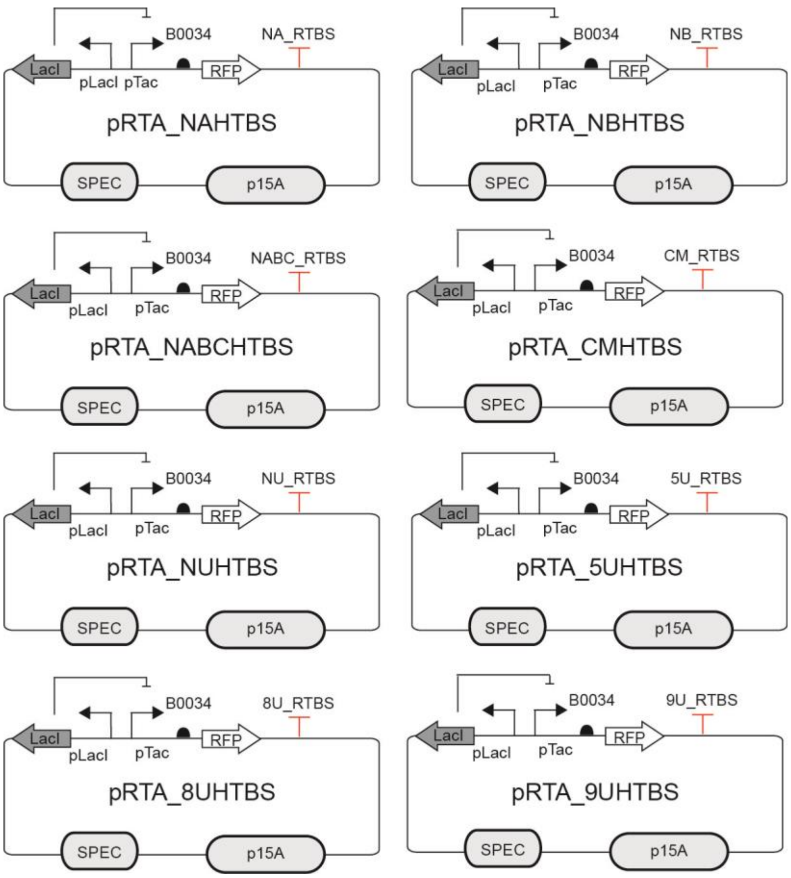


**Supplementary Figure 15: HIV reverse transcriptase activity measurements plasmids maps.** These plasmids were used for experiments depicted in Figure 1(d-e). Part sequences are provided in Supplementary Tables 1-4. Parts beginning with "B" are from the Registry of Standard Biological Parts. The HTBS variants are named as in Supplementary Table 1.

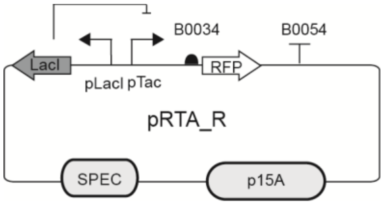


**Supplementary Figure 16: HIV reverse transcriptase control measurements plasmid map.** The control uses the strong BBa_B0054 terminator, as opposed to an HTBS part containing the HIVRT recognition hairpin. This plasmid was used to generate the data in Figure 1e. Part sequences are provided in Supplementary Tables 1-4. Parts beginning with "B" are from the Registry of Standard Biological Parts.

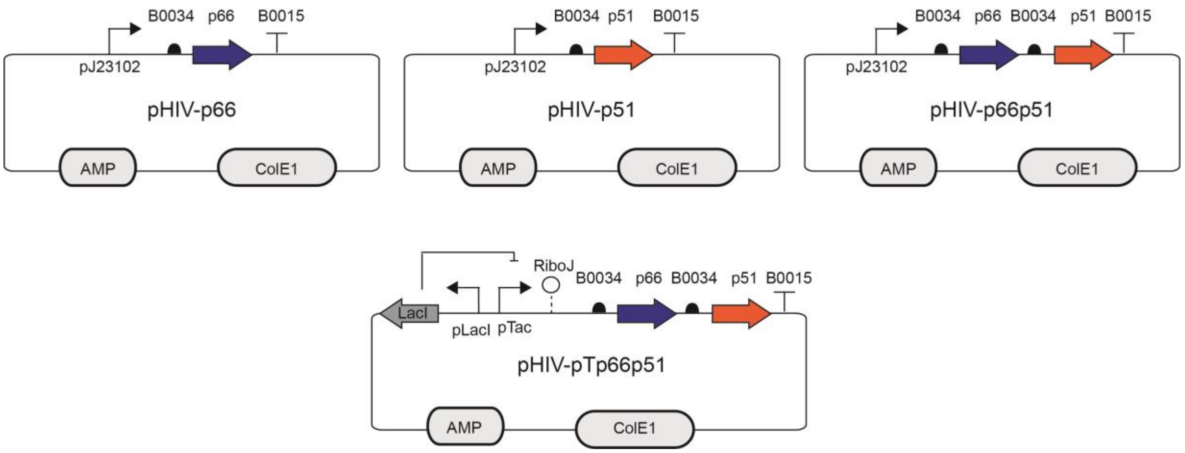


**Supplementary Figure 17: HIV reverse transcriptase plasmids maps**. The plasmids (pHIV-p66, pHIV-p51 and pHIV-p66p51) were used in generate data shown in Figure 1-2, while plasmid pHIV-pTp66p51 ("initiator" plasmid) was used for data shown in Figure 3-5. Part sequences are provided in in Supplementary Tables 1-4. Parts beginning with "B" and promoter J23102 are from the Registry of Standard Biological Parts.

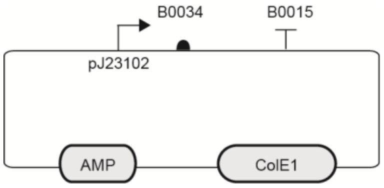


**Supplementary Figure 18: Control plasmid for HIVRT expression.** This plasmid is identical to that used to express the HIVRT genes and is used as a control. Parts beginning with "B" and promoter J23102 are from the Registry of Standard Biological Parts.

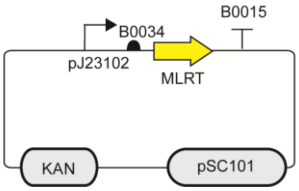


**Supplementary Figure 19: Murine leukemia reverse transcriptase plasmid map.** This plasmid is referred to as the "Amplifier" in Figure 4a. Part sequences are provided in Supplementary Tables 1-4. Parts beginning with "B" and promoter J23102 are from the Registry of Standard Biological Parts.

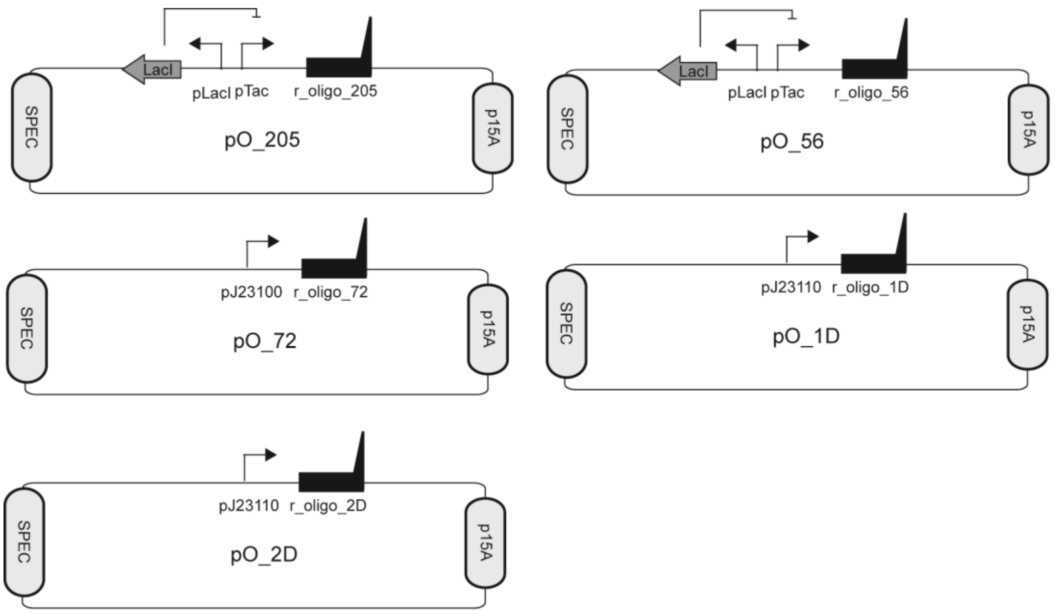


**Supplementary Figure 20: Plasmids used for different combinations of r_oligo genes.** These plasmids were used in Figure 2-3 and Supplementary Figure 1-4. Part sequences are provided in Supplementary Tables 1-4. Parts beginning with "B" are from the Registry of Standard Biological Parts. r_oligo_###, ### represents the number of nucleotides included in the ssDNA or the DNA structure assemble from this part.

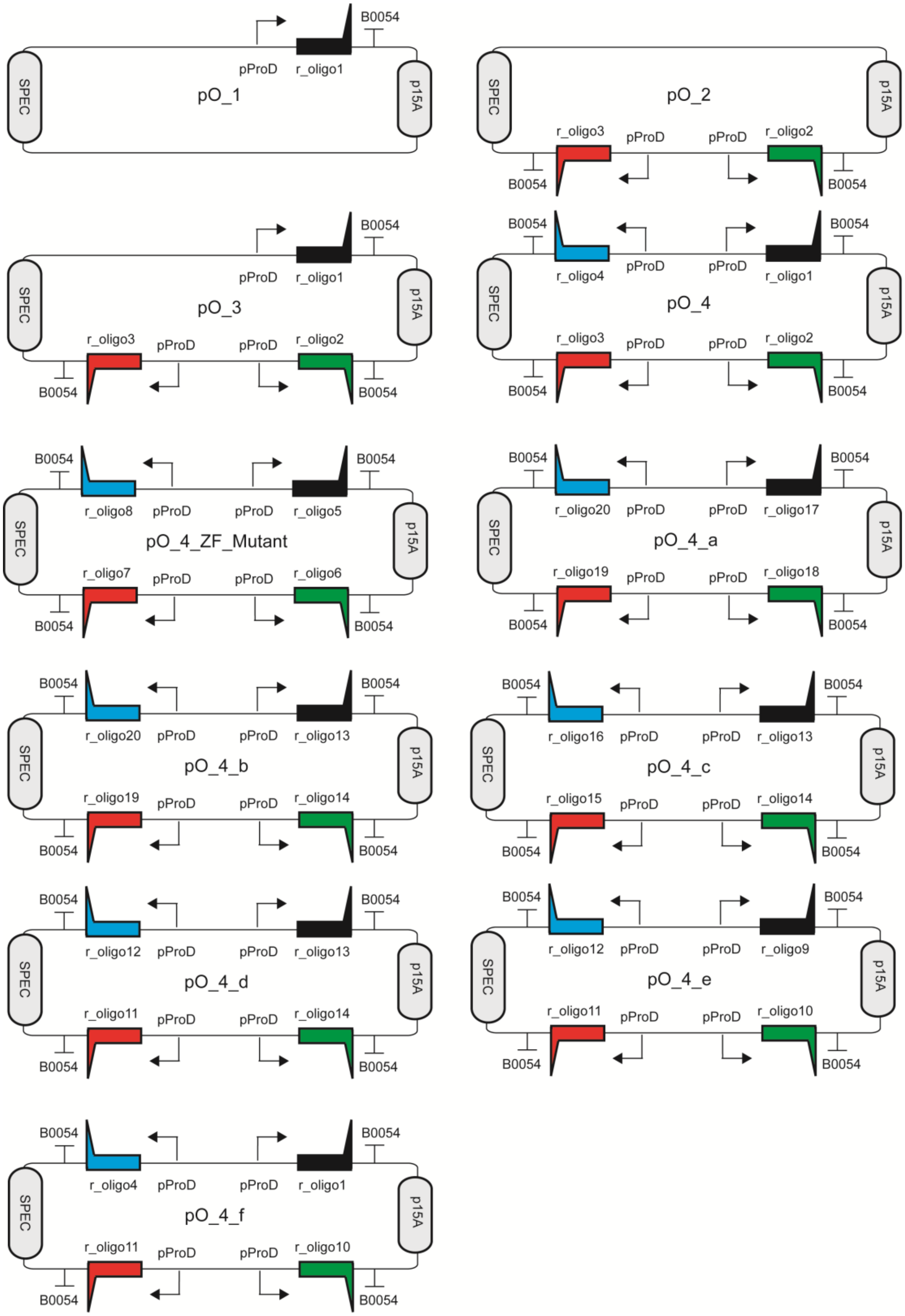


**Supplementary Figure 21: Plasmids used for different combinations of r_oligo genes.** These plasmids were used in Figure 4-5. Part sequences are provided in Supplementary Tables 1-4. Parts beginning with "B" are from the Registry of Standard Biological Parts.

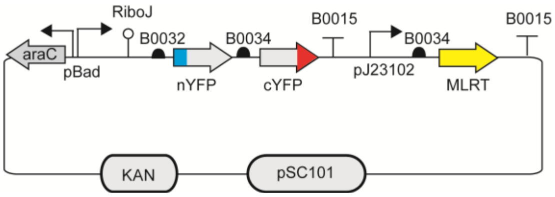


**Supplementary Figure 22: Plasmid map used for the split GFP assay shown in Figure 5**.This plasmid include the split YFP parts and the MLRT part. Part sequences are provided in Supplementary Tables 3. Parts beginning with “B” are from the Registry of Standard Biological.

**Supplementary Table 1:** HTBSs sequences

| Name | Sequence |
|---|---|
| NA_HTBS | AAAAAAAACGUGGCGCCCGAACAGGGACGGAUCCGCCCGGAUAAUCAGACUUUUAAUCUGAGGGUC CAGGGUUCAAGUCCCUGUUCGGGCGCCACGUUUUUUUU |
| NB_HTBS | AAAAAAAACGUGGCGCCCGAACAGGGACGGAUCCGCCCGGAUAGCUCAGUCGGUAGAGCAUCAGAC UUUUAAUCUGAGUCCCUGUUCGGGCGCCACGUUUUUUUU |
| NABC_HTBS | AAAAAAAACGUGGCGCCCGAACAGGGACUCGUGGAAUGUCCCUGUUCGGGCGCCACGUUUUUUUU |
| C*_HTBS | AAAAAAAACGUGGCGCCCGAACAGGGACGGAUAGCUCAGUCGGUAGAGCAUCAGACUUUUAAUCUG AGGGUCCAGGGUUCAAGUCCCUGUUCGGGCGCCACGUUUUUUUU |
| NU_HTBS | AAAAAAAACGUGGCGCCCGAACAGGGACGGAUCCGCCCGGAUAGCUCAGUCGGUAGAGCAUCAGAC UUUUAAUCUGAGGGUCCAGGGUUCAAGUCCCUGUUCGGGCGCCACG |
| 5U_HTBS | AAAAACGUGGCGCCCGAACAGGGACGGAUCCGCCCGGAUAGCUCAGUCGGUAGAGCAUCAGACUUU UAAUCUGAGGGUCCAGGGUUCAAGUCCCUGUUCGGGCGCCACGUUUUU |
| 8U_HTBS | AAAAAAAACGUGGCGCCCGAACAGGGACGGAUCCGCCCGGAUAGCUCAGUCGGUAGAGCAUCAGAC UUUUAAUCUGAGGGUCCAGGGUUCAAGUCCCUGUUCGGGCGCCACGUUUUUUUU |
| 9U_HTBS | AAAAAAAAACGUGGCGCCCGAACAGGGACGGAUCCGCCCGGAUAGCUCAGUCGGUAGAGCAUCAGA CUUUUAAUCUGAGGGUCCAGGGUUCAAGUCCCUGUUCGGGCGCCACGUUUUUUUUU |

**Supplementary Table 2:** ssDNAs sequences

| Name | Sequence |
|---|---|
| r_oligo205 | TAAGCTTTGGAACCGTACTGGAACTGCGGGGACAGGATGTCCCAAGCGAACGGCAGCGGACCACCTTTGGTAACTTTCAGTTTAGCGGTCTGGGTACCTTCGTACGGACGACCTTCACCTTCACCTTCGATTTCGAACTCGTGACCGTTAACGGAACCGGACAGGAGGCTAGCTACAACGAGTCCCAAGAATTGTGAGCGCTCAC |
| r_oligo56 | AACGGAACCGGACAGGAGGCTAGCTACAACGAGTCCCAAGATTGTGAGCGCTCAC |
| r_oligo72 | CGCGGGTTATAGTGCAGTCGTGATTCGTGCGCAGGCTATGTGTCTCATCTTAAATGTTTAGGGAATGGTTTT |
| r_oligo1D | TGACCGATTGGACAGGCCTGTGGTCACCAATC |
| r_olIgo2D | TGGACCGATTGATAAGACTTCAGGCCTGAAGTGGTCCACTTATCAATC |
| r_oligo1 | TGCGCAATCCCGCACCCGCTTGAGCACGCCAACATCACCGTATTT |
| r_oligo2 | GCGGCTAGCAGAGCATTCGGGAAAGGTGTGACTATGGCTGTATTT |
| r_oligo3 | GGCGTGCTCACACACCTTTCGGATTGCGCATGCTAGCCGCTATTT |
| r_oligo4 | CGGTGATGTTCAGCCATAGTAGCGGGTGCGCCGAATGCTCTATTT |
| r_oligo5 | TGCGCAATCCCGCTGAGGCTTGAGCACGCCAACATCACCGTATTT |
| r_oligo6 | GCGGCTAGCAGAGCATTCGGGCCCGCTGAGACTATGGCTGTATTT |
| r_oligo7 | GGCGTGCTCACTCAGCGGGCGGATTGCGCATGCTAGCCGCTATTT |
| r_oligo8 | CGGTGATGTTCAGCCATAGTAGCCTCAGCGCCGAATGCTCTATTT |
| r_oligo9 | TGCGCAATCCCGCACCCGCTTGAGCACGCCTATTT |
| r_oligo10 | GAGCATTCGGGAAAGGTGTGACTATGGCTGTATTT |
| r_oligo11 | GGCGTGCTCACACACCTTTCGGATTGCGCATATTT |
| r_oligo12 | CAGCCATAGTAGCGGGTGCGCCGAATGCTCTATTT |
| r_oligo13 | CGCACCCGCTTGAGCACGCCTATTT |
| r_oligo14 | GAAAGGTGTGACTATGGCTGTATTT |
| r_oligo15 | GGCGTGCTCACACACCTTTCTATTT |
| r_oligo16 | CAGCCATAGTAGCGGGTGCGTATTT |
| r_oligo17 | CGCACCCGCTTATTT |
| r_oligo18 | GAAAGGTGTGTATTT |
| r_oligo19 | CACACCTTTCTATTT |
| r_oligo20 | AGCGGGTGCGTATTT |

**Supplementary Table 3:** Reverse transcriptase sequences

| Name | Sequence |
|---|---|
| p66 | ATGCCGATTAGCCCGATTGAAACCGTTCCGGTTAAACTGAAACCGGGTATGGATGGTCCGAAAGTTAAACAGTGGCCTCTGAC<br>CGAAGAAAAAATCAAAGCACTGGTTGAAATCTGCACCGAGATGGAAAAAGAAGGCAAAATTAGCAAAATCGGTCCGGAAAATC<br>CGTATAATACACCGGTTTTTGCCATTAAGAAAAAAGATAGCACCAAATGGCGCAAACTGGTGGATTTTCGTGAACTGAATAAA<br>CGCACCCAGGATTTTTGGGAAGTTCAGCTGGGTATTCCGCATCCGGCAGGTCTGAAACAGAAAAAAAGCGTTACCGTTCTGGA<br>TGTTGGTGATGCATATTTTAGCGTTCCGCTGGATAAAGATTTCCGTAAATATACCGCATTTACCATCCCGAGCATTAATAACG<br>AAACACCGGGTATTCGCTATCAGTATAATGTTCTGCCGCAGGGTTGGAAAGGTAGTCCGGCAATTTTTCAGTGTAGCATGACC<br>AAAATTCTGGAACCGTTTCGTAAACAGAATCCGGATATTGTGATCTACCAGTATATGGATGATCTGTATGTTGGTAGCGATCT<br>GGAAATTGGTCAGCATCGTACCAAAATTGAAGAACTGCGTCAGCATCTGCTGCGTTGGGGTTTTACCACACCGGATAAAAAAC<br>ATCAGAAAGAACCGCCTTTTCTGTGGATGGGTTATGAACTGCATCCGGATAAATGGACCGTTCAGCCGATTGTTCTGCCGGAA<br>AAAGATAGCTGGACCGTTAATGATATTCAGAAACTGGTGGGTAAACTGAATTGGGCAAGCCAGATTTATGCCGGTATTAAAGT<br>TCGTCAGCTGTGTAAACTGCTGCGTGGCACCAAAGCACTGACCGAAGTTGTTCCGCTGACAGAAGAAGCAGAACTGGAACTGG<br>CAGAAAATCGTGAAATTCTGAAAGAACCGGTTCACGGCGTTTATTATGATCCGAGCAAAGATCTGATTGCCGAAATTCAGAAA<br>CAGGGTCAGGGTCAGTGGACCTATCAGATTTATCAAGAACCGTTTAAAAACCTGAAAACCGGCAAATATGCACGTATGAAAGG<br>TGCACATACCAACGATGTTAAACAGCTGACCGAAGCAGTTCAGAAAATTGCAACCGAAAGCATTGTGATTTGGGGTAAAACCC<br>CGAAATTCAAACTGCCGATTCAGAAAGAAACCTGGGAAGCATGGTGGACCGAATATTGGCAGGCAACCTGGATTCCGGAATGG<br>GAATTTGTTAATACCCCTCCGCTGGTTAAACTGTGGTATCAGCTGGAAAAAGAACCGATTATTGGTGCCGAAACCTTTTATGT<br>TGATGGTGCAGCCAATCGTGAAACCAAACTGGGTAAAGCAGGTTATGTTACCGATCGTGGTCGTCAGAAAGTGGTGCCGCTGA<br>CCGATACCACCAATCAGAAAACCGAACTGCAGGCAATTCATCTGGCACTGCAGGATAGCGGTCTGGAAGTTAATATTGTTACC<br>GATAGCCAGTATGCCCTGGGTATTATTCAGGCACAGCCGGATAAAAGCGAAAGCGAACTGGTTAGCCAGATTATTGAACAGCT<br>GATCAAAAAAGAAAAAGTGTACCTGGCATGGGTTCCGGCACATAAAGGTATTGGTGGTAATGAACAGGTTGATGGTCTGGTTA<br>GCGCAGGTATTCGTAAAGTTCTGTAA |
| p51 | ATGCCGATTAGCCCGATTGAAACCGTTCCGGTTAAACTGAAACCGGGTATGGATGGTCCGAAAGTTAAACAGTGGCCTCTGAC<br>CGAAGAAAAAATCAAAGCACTGGTTGAAATCTGCACCGAGATGGAAAAAGAAGGCAAAATTAGCAAAATCGGTCCGGAAAATC<br>CGTATAATACACCGGTTTTTGCCATTAAGAAAAAAGATAGCACCAAATGGCGCAAACTGGTGGATTTTCGTGAACTGAATAAA<br>CGCACCCAGGATTTTTGGGAAGTTCAGCTGGGTATTCCGCATCCGGCAGGTCTGAAACAGAAAAAAAGCGTTACCGTTCTGGA<br>TGTTGGTGATGCATATTTTAGCGTTCCGCTGGATAAAGATTTCCGTAAATATACCGCATTTACCATCCCGAGCATTAATAACG<br>AAACACCGGGTATTCGCTATCAGTATAATGTTCTGCCGCAGGGTTGGAAAGGTAGTCCGGCAATTTTTCAGTGTAGCATGACC<br>AAAATTCTGGAACCGTTTCGTAAACAGAATCCGGATATTGTGATCTACCAGTATATGGATGATCTGTATGTTGGTAGCGATCT<br>GGAAATTGGTCAGCATCGTACCAAAATTGAAGAACTGCGTCAGCATCTGCTGCGTTGGGGTTTTACCACACCGGATAAAAAAC<br>ATCAGAAAGAACCGCCTTTTCTGTGGATGGGTTATGAACTGCATCCGGATAAATGGACCGTTCAGCCGATTGTTCTGCCGGAA<br>AAAGATAGCTGGACCGTTAATGATATTCAGAAACTGGTGGGTAAACTGAATTGGGCAAGCCAGATTTATGCCGGTATTAAAGT<br>TCGTCAGCTGTGTAAACTGCTGCGTGGCACCAAAGCACTGACCGAAGTTGTTCCGCTGACAGAAGAAGCAGAACTGGAACTGG<br>CAGAAAATCGTGAAATTCTGAAAGAACCGGTTCACGGCGTTTATTATGATCCGAGCAAAGATCTGATTGCCGAAATTCAGAAA<br>CAGGGTCAGGGTCAGTGGACCTATCAGATTTATCAAGAACCGTTTAAAAACCTGAAAACCGGCAAATATGCACGTATGAAAGG<br>TGCACATACCAACGATGTTAAACAGCTGACCGAAGCAGTTCAGAAAATTGCAACCGAAAGCATTGTGATTTGGGGTAAAACCC<br>CGAAATTCAAACTGCCGATTCAGAAAGAAACCTGGGAAGCATGGTGGACCGAATATTGGCAGGCAACCTGGATTCCGGAATGG<br>GAATTTGTTAATACCCCTCCGCTGGTTAAACTGTGGTATCAGCTGGAAAAAGAACCGATTATTGGTGCCGAAACCTTTTAA |
| MLRT | ATGGGTCATAATCATAATCATAATCATAATCATAATCACAACGGTGGAGATGACGATGACAAGGGTGGTCGACAAGCTTGGAT<br>CCCTGCAGGCCTCAGGGCCCGATCGATGGGACCAATGGGGCAGCCCCTGCAAGTGTTGACCCTAAATATAGAAGATGAGTATC<br>GGCTACATGAGACCTCAAAAGAGCCAGATGTTTCTCTAGGGTCCACATGGCTGTCTGATTTTCCTCAGGCCTGGGCGGAAACC<br>GGGGGCATGGGACTGGCAGTTCGCCAAGCTCCTCTGATCATACCTCTGAAAGCAACCTCTACCCCCGTGTCCATAAAACAATA<br>CCCCATGTCACAAGAAGCCAGACTGGGGATCAAGCCCCACATACAGAGACTGTTGGACCAGGGAATACTGGTACCCTGCCAGT<br>CCCCCTGGAACACGCCCCTGCTACCCGTTAAGAAACCAGGGACTAATGATTATAGGCCTGTCCAGGATCTGAGAGAAGTCAAC<br>AAGCGGGTGGAAGACATCCACCCCACCGTGCCCAACCCTTACAACCTCTTGAGCGGGCTCCCACCGTCCCACCAGTGGTACAC<br>TGTGCTTGATTTAAAGGATGCCTTTTTCTGCCTGAGACTCCACCCCACCAGTCAGCCTCTCTTCGCCTTTGAGTGGAGAGATC<br>CAGAGATGGGAATCTCAGGACAATTGACCTGGACCAGACTCCCACAGGGTTTCAAAAACAGTCCCACCCTGTTTGATGAGGCA<br>CTGCACAGAGACCTAGCAGACTTCCGGATCCAGCACCCAGACTTGATCCTGCTACAGTACGTGGATGACTTACTGCTGGCCGC<br>CACTTCTGAGCTAGACTGCCAACAAGGTACTCGGGCCCTGTTACAAACCCTAGGGAACCTCGGGTATCGGGCCTCGGCCAAGA<br>AAGCCCAAATTTGCCAGAAACAGGTCAAGTATCTGGGGTATCTTCTAAAAGAGGGTCAGAGATGGCTGACTGAGGCCAGAAAA<br>GAGACTGTGATGGGGCAGCCTACTCCGAAGACCCCTCGACAACTAAGGGAGTTCCTAGGGACGGCAGGCTTCTGTCGCCTCTG<br>GATCCCTGGGTTTGCAGAAATGGCAGCCCCCTTGTACCCTCTCACCAAAACGGGGACTCTGTTTAATTGGGGCCCAGACCAAC<br>AAAAGGCCTATCAAGAAATCAAGCAAGCTCTTCTAACTGCCCCAGCCCTGGGGTTGCCAGATTTGACTAAGCCCTTTGAACTC<br>TTTGTCGACGAGAAGCAGGGCTACGCCAAAGGTGTCCTAACGCAAAAACTGGGACCTTGGCGTCGGCCGGTGGCCTACCTGTC<br>CAAAAAGCTAGACCCAGTAGCAGCTGGGTGGCCCCCTTGCCTACGGATGGTAGCAGCCATTGCCGTACTGACAAAGGATGCAG<br>GCAAGCTAACCATGGGACAGCCACTAGTCATTCTGGCCCCCCATGCAGTAGAGGCACTAGTCAAACAACCCCCCGACCGCTGG<br>CTTTCCAACGCCCGGATGACTCACTATCAGGCCTTGCTTTTGGACACGGACCGGGTCCAGTTCGGACCGGTGGTAGCCCTGAA<br>CCCGGCTACGCTGCTCCCACTGCCTGAGGAAGGGCTGCAACACAACTGCCTTGATATCCTGGCCGAAGCCCACGGAACCCGAC |

CCGACCTAACGGACCAGCCGCTCCCAGACGCCGACCACACCTGGTACACGGATGGAAGCAGTCTCTTACAAGAGGGACAGCGT
AAGGCGGGAGCTGCGGTGACCACCGAGACCGAGGTAATCTGGGCTAAAGCCCTGCCAGCCGGGACATCCGCTCAGCGGGCTGA
ACTGATAGCACTCACCCAGGCCCTAAAGATGGCAGAAGGTAAGAAGCTAAATGTTTATACTGATAGCCGTTATGCTTTTGCTA
CTGCCCATATCCATGGAGAAATATACAGAAGGCGTGGGTTGCTCACATCAGAAGGCAAAGAGATCAAAAATAAAGACGAGATC
TTTAAATGA

---

**Supplementary Table 4:** Genetic part sequences

| Name | Sequence |
|---|---|
| BBa_J23102 | TTGACAGCTAGCTCAGTCCTAGGTACTGTGCTAGC |
| BBa_J23110 | TTTACGGCTAGCTCAGTCCTAGGTACAATGCTAGC |
| proD | CACAGCTAACACCACGTCGTCCCTATCTGCTGCCCTAGGTCTATGAGTGGTTGCTGGATAACTTTACGGGCATGCATAA<br>GGCTCGTATAATATATTCAGGGAGACCACAACGGTTTCCCTCTACAAATAATTTTGTTTAACTTT |
| pLacI[a] | GCGGCGCGCCATCGAATGGCGCAAAACCTTTCGCGGTATGGCATGATAGCGCCCGGAAGAGAGTCAATTCAGGGTGGTG<br>AAT |
| pTac[a] | TGTTGACAATTAATCATCGGCTCGTATAATGTGTGGAATTGTGAGCGCTCACAATT |
| LacI[a] | ATGAAACCAGTAACGTTATACGATGTCGCAGAGTATGCCGGTGTCTCTTATCAGACCGTTTCCCGCGTGGTGAACCAGG<br>CCAGCCACGTTTCTGCGAAAACGCGGGAAAAAGTGGAAGCGGCGATGGCGGAGCTGAATTACATTCCCAACCGCGTGGC<br>ACAACAACTGGCGGGCAAACAGTCGTTGCTGATTGGCGTTGCCACCTCCAGTCTGGCCCTGCACGCGCCGTCGCAAATT<br>GTCGCGGCGATTAAATCTCGCGCCGATCAACTGGGTGCCAGCGTGGTGGTGTCGATGGTAGAACGAAGCGGCGTCGAAG<br>CCTGTAAAGCGGCGGTGCACAATCTTCTCGCGCAACGCGTCAGTGGGCTGATCATTAACTATCCGCTGGATGACCAGGA<br>TGCCATTGCTGTGGAAGCTGCCTGCACTAATGTTCCGGCGTTATTTCTTGATGTCTCTGACCAGACACCCATCAACAGT<br>ATTATTTTCTCCCATGAGGACGGTACGCGACTGGGCGTGGAGCATCTGGTCGCATTGGGTCACCAGCAAATCGCGCTGT<br>TAGCGGGCCCATTAAGTTCTGTCTCGGCGCGTCTGCGTCTGGCTGGCTGGCATAAATATCTCACTCGCAATCAAATTCA<br>GCCGATAGCGGAACGGGAAGGCGACTGGAGTGCCATGTCCGGTTTTCAACAAACCATGCAAATGCTGAATGAGGGCATC<br>GTTCCCACTGCGATGCTGGTTGCCAACGATCAGATGGCGCTGGGCGCAATGCGCGCCATTACCGAGTCCGGGCTGCGCG<br>TTGGTGCGGATATCTCGGTAGTGGGATACGACGATACCGAAGATAGCTCATGTTATATCCCGCCGTTAACCACCATCAA<br>ACAGGATTTTCGCCTGCTGGGGCAAACCAGCGTGGACCGCTTGCTGCAACTCTCTCAGGGCCAGGCGGTGAAGGGCAAT<br>CAGCTGTTGCCAGTCTCACTGGTGAAAAGAAAAACCACCCTGGCGCCCAATACGCAAACCGCCTCTCCCCGCGCGTTGG<br>CCGATTCATTAATGCAGCTGGCACGACAGGTTTCCCGACTGGAAAGCGGGCAGTGAGCGCAACGCAATTAATGTAAGTT<br>AGCGCGAATTGGCCGAC |
| pBAD-araC | AGTTATGACAACTTGACGGCTACATCATTCACTTTTTCTTCACAACCGGCACGGAACTCGCTCGGGCTGGCCCCGGTGC<br>ATTTTTTAAATACCCGCGAGAAATAGAGTTGATCGTCAAAACCAACATTGCGACCGACGGTGGCGATAGGCATCCGGGT<br>GGTGCTCAAAAGCAGCTTCGCCTGGCTGATACGTTGGTCCTCGCGCCAGCTTAAGACGCTAATCCCTAACTGCTGGCGG<br>AAAAGATGTGACAGACGCGACGGCGACAAGCAAACATGCTGTGCGACGCTGGCGATATCAAAATTGCTGTCTGCCAGGT<br>GATCGCTGATGTACTGACAAGCCTCGCGTACCCGATTATCCATCGGTGGATGGAGCGACTCGTTAATCGCTTCCATGCG<br>CCGCAGTAACAATTGCTCAAGCAGATTTATCGCCAGCAGCTCCGAATAGCGCCCTTCCCCTTGCCCGGCGTTAATGATT<br>TGCCCAAACAGGTCGCTGAAATGCGGCTGGTGCGCTTCATCCGGGCGAAAGAACCCCGTATTGGCAAATATTGACGGCC<br>AGTTAAGCCATTCATGCCAGTAGGCGCGCGGACGAAAGTAAACCCACTGGTGATACCATTCGCGAGCCTCCGGATGACG<br>ACCGTAGTGATGAATCTCTCCTGGCGGGAACAGCAAAATATCACCCGGTCGGCAAACAAATTCTCGTCCCTGATTTTTC<br>ACCACCCCCTGACCGCGAATGGTGAGATTGAGAATATAACCTTTCATTCCCAGCGGTCGGTCGATAAAAAAATCGAGAT<br>AACCGTTGGCCTCAATCGGCGTTAAACCCGCCACCAGATGGGCATTAAACGAGTATCCCGGCAGCAGGGGATCATTTTG<br>CGCTTCAGCCATACTTTTCATACTCCCGCCATTCAGAGAAGAAACCAATTGTCCATATTGCATCAGACATTGCCGTCAC<br>TGCGTCTTTTACTGGCTCTTCTCGCTAACCAAACCGGTAACCCCGCTTATTAAAAGCATTCTGTAACAAAGCGGGACCA<br>AAGCCATGACAAAAACGCGTAACAAAAGTGTCTATAATCACGGCAGAAAAGTCCACATTGATTATTTGCACGGCGTCAC<br>ACTTTGCTATGCCATAGCATTTTTATCCATAAGATTAGCGGATCCTACCTGACGCTTTTTATCGCAACTCTCTACTGTT<br>TCTCC |
| BBa_B0015 | CCAGGCATCAAATAAAACGAAAGGCTCAGTCGAAAGACTGGGCCTTTCGTTTTATCTGTT<br>GTTTGTCGGTGAACGCTCTCTACTAGAGTCACACTGGCTCACCTTCGGGTGGGCCTTTCTGCG TTTATA |
| BBa_B0054 | ATTAGCAGAAAGTCAAAAGCCTCCGACCGGAGGCTTTTGACTAAAACTTCCCTTGGGGTTATCATTGGG |
| RiboJ | CTGTCACCGGATGTGCTTTCCGGTCTGATGAGTCCGTGAGGACGAAACAG |
| B0034 | AAAGAGGAGAAA |
| B0032 | TCACACAGGAAAG |
| RFP | ATGGCTTCCTCCGAAGACGTTATCAAAGAGTTCATGCGTTTCAAAGTTCGTATGGAAGGTTCCGTTAACGGTCACGAGT |

| | |
|---|---|
| | TCGAAATCGAAGGTGAAGGTGAAGGTCGTCCGTACGAAGGTACCCAGACCGCTAAACTGAAAGTTACCAAAGGTGGTCC<br>GCTGCCGTTCGCTTGGGACATCCTGTCCCCGCAGTTCCAGTACGGTTCCAAAGCTTACGTTAAACACCCGGCTGACATC<br>CCGGACTACCTGAAACTGTCCTTCCCGGAAGGTTTCAAATGGGAACGTGTTATGAACTTCGAAGACGGTGGTGTTGTTA<br>CCGTTACCCAGGACTCCTCCCTGCAAGACGGTGAGTTCATCTACAAAGTTAAACTGCGTGGTACCAACTTCCCGTCCGA<br>CGGTCCGGTTATGCAGAAAAAAACCATGGGTTGGGAAGCTTCCACCGAACGTATGTACCCGGAAGACGGTGCTCTGAAA<br>GGTGAAATCAAAATGCGTCTGAAACTGAAAGACGGTGGTCACTACGACGCTGAAGTTAAAACCACCTACATGGCTAAAA<br>AACCGGTTCAGCTGCCGGGTGCTTACAAAACCGACATCAAACTGGACATCACCTCCCACAACGAAGACTACACCATCGT<br>TGAACAGTACGAACGTGCTGAAGGTCGTCACTCCACCGGTGCTTAATAA |
| GFP | ATGCGTAAAGGAGAAGAACTTTTCACTGGAGTTGTCCCAATTCTTGTTGAATTAGATGGTGATGTTAATGGGCACAAAT<br>TTTCTGTCAGTGGAGAGGGTGAAGGTGATGCAACATACGGAAAACTTACCCTTAAATTTATTTGCACTACTGGAAAACT<br>ACCTGTTCCATGGCCAACACTTGTCACTACTTTCGGTTATGGTGTTCAATGCTTTGCGAGATACCCAGATCATATGAAA<br>CAGCATGACTTTTTCAAGAGTGCCATGCCCGAAGGTTATGTACAGGAAAGAACTATATTTTTCAAAGATGACGGGAACT<br>ACAAGACACGTGCTGAAGTCAAGTTTGAAGGTGATACCCTTGTTAATAGAATCGAGTTAAAAGGTATTGATTTTAAAGA<br>AGATGGAAACATTCTTGGACACAAATTGGAATACAACTATAACTCACACAATGTATACATCATGGCAGACAAACAAAAG<br>AATGGAATCAAAGTTAACTTCAAAATTAGACACAACATTGAAGATGGAAGCGTTCAACTAGCAGACCATTATCAACAAA<br>ATACTCCAATTGGCGATGGCCCTGTCCTTTTACCAGACAACCATTACCTGTCCACACAATCTGCCCTTTCGAAAGATCC<br>CAACGAAAAGAGAGACCACATGGTCCTTCTTGAGTTTGTAACAGCTGCTGGGATTACACATGGCATGGATGAACTATAC<br>AAATAATAA3’ |
| nYFP | ATGTATCATCACCATCACCATCACACTAGACCGGGTGAAAAACCGTATGCATGTCCTGAATGTGGTAAAAGCTTTAGCC<br>AGCGTGCAAATCTGCGTGCACATCAGCGTACCCATACAGGTGAAAAGCCTTATAAATGCCCAGAATGCGGCAAAAGCTT<br>TTCACGTAGCGATCATCTGACCACCCATCAGCGCACACATACTGGCGAGAAACCTTACAAATGTCCAGAGTGTGGTAAA<br>TCATTTAGCCGTAGTGATGTTCTGGTTCGTCATCAGAGAACCCACACGGGTGGTGGTAGCGGTGGTGGTTCAGGTGGTA<br>GTACTAGAGTGAGCAAGGGCGAGGAGCTGTTCACCGGGGTGGTGCCCATCCTGGTCGAGCTGGACGGCGACGTAAACGG<br>CCACAAGTTCAGCGTGTCCGGCGAGGGCGAGGGCGATGCCACCTACGGCAAGCTGACCCTGAAGTTCATCTGCACCACC<br>GGCAAGCTGCCCGTGCCCTGGCCCACCCTCGTGACCACCTTCGGCTACGGCCTGATGTGCTTCGCCCGCTACCCCGACC<br>ACATGAAGCAGCACGACTTCTTCAAGTCCGCCATGCCCGAAGGCTACGTCCAGGAGCGCACCATCTTCTTCAAGGACGA<br>CGGCAACTACAAGACCCGCGCCGAGGTGAAGTTCGAGGGCGACACCCTGGTGAACCGCATCGAGCTGAAGGGCATCGAC<br>TTCAAGGAGGACGGCAACATCCTGGGGCACAAGCTGGAGTACAACTACAACAGCCACAACGTCTATATCATGGCCGACA<br>AGCAGAAGAACGGCATCAAGGTGAACTTCAAGATCCGCCACAACATCTAA |
| cYFP | ATGGCCGACAAGCAGAAGAACGGCATCAAGGTGAACTTCAAGATCCGCCACAACATCGAGGACGGCAGCGTGCAGCTCG<br>CCGACCACTACCAGCAGAACACCCCCATCGGCGACGGCCCCGTGCTGCTGCCCGACAACCACTACCTGAGCTACCAGTC<br>CGCCCTGAGCAAAGACCCCAACGAGAAGCGCGATCACATGGTCCTGCTGGAGTTCGTGACCGCCGCCGGGATCACTCTC<br>GGCATGGACGAGCTGTACAAGGGTGGCTCTGGCGGTGGGAGTGGGGGAAGCACTAGACCGGGTGAAAAACCGTATGCAT<br>GTCCGGTTGAAAGCTGTGATCGTCGTTTTAGCCGTAGTGATGAACTGACCCGTCATATTCGTATTCATACAGGTCAGAA<br>ACCGTTTCAGTGTCGTATTTGCATGCGTAATTTTAGCCGTTCAGATCATCTGACCACCCATATTCGTACCCATACTGGC<br>GAAAAACCGTTTGCCTGTGATATTTGTGGTCGTAAATTTGCACGTTCCGATGAACGTAAACGCCATACCAAAATTCATA<br>CGGGTGGTGGTAGCGGTGGTGGTTCAGGTGGTAGTACTAGACACCACCACCACCACCACTAA |

*a. pLacI, pTac and LacI sequences are based on the pEXT20 plasmid(addgene)*

**Supplementary Table 5:** Oligo sequences used during the short ssDNA sequencing assay

| Name | Sequence |
|---|---|
| CL53 | CGACGCTCTTC-ddC (ddC = dideoxycytidine) |
| CL73 | [Phosphate]GGAAGAGCGTCGTGTAGGGAAAGAG*T*G*T*A (* = phosphothioate linkage) |
| CL78 | [Phosphate]AGATCGGAAG[C3Spacer]10[TEG-biotin] (TEG = triethylene glycol spacer) |
| | CAAGCAGAAGACGGCATACGAGATCCTGCGATTGTTTTTCTTTGTTTCTTTTTCTTGTCTTTCTTTG |
| P7L | ATGTGACTGGAGTTCAGACGTGT |
| P5 | ACGCTCTTCCCGCGGGTTAT |
| SEQ | CAAGCAGAAGACGGCATACGAGATCCTGCGA |

**Supplementary Methods**

**Materials**

**Strains and media**. *Escherichia coli* DH10β (MC1061 F-endA1 recA1 galE15 galK16 nupG rpsL ΔlacX74 Φ80lacZΔM15 araD139 Δ(ara,leu)7697 mcrA Δ(mrr-hsdRMS-mcrBC) λ-) was used for all manipulations and assays except for the terminator measurements, which were performed in *E. coli* DH5α (*fhuA2 lac(del)U169 phoA glnV44 Φ80' lacZ(del)M15 gyrA96 recA1 relA1 endA1 thi-1 hsdR17*). Cells were grown in LB Miller Broth (10 g/l tryptone, 5 g/l yeast extract, 10 g/l NaCl, Fisher Scientific. Ampicillin (100 µg per ml, Affymetrix cat. #11259-5), kanamycin (50 µg per ml, Gold Bio cat. #K-120-5) and/or Spectinomycin (100 µg per ml, MP Biomedicals cat. #021 5899305) were used where appropriate. Isopropyl β-D-1-thiogalactopyranoside (IPTG, Roche cat. #10 745 740 001) or L-arabinose (L-ara, USB Corporation # 5328 37 0) were used as inducers.

The following materials were used for the sequencing of short ssDNA experiment: water, HPLC grade (Sigma-Aldrich, cat. no. 270733), NaCl solution, 5 M (Sigma-Aldrich, cat. no. S5150-1L), EDTA solution, pH 8.0, 0.5 M (AppliChem, cat. no. A4892,1000), SDS solution, 20% (wt/vol; Ambion, cat. no. AM9820), SSC buffer, 20× (Ambion, cat. no. AM9770), Tris-HCl solution, pH 8.0, 1 M (AppliChem, cat. no. A4577,0500), Tween 20 (Sigma-Aldrich, cat. no. P5927-100ML), Bead-binding and wash buffers (Reagent Setup), *A. fulgidus* uracil-DNA glycosylase (Afu UDG, NEB, cat. no. M0279L), Endonuclease VIII (NEB, cat. no. M0299L), FastAP thermosensitive alkaline phosphatase (Thermo Scientific, cat. no. EF0651), CircLigase II ssDNA ligase (Epicentre, cat. no. CL9025K), including 10× reaction buffer and 50 mM MnCl2 solution, Dynabeads MyOne streptavidin C1 (Life Technologies, cat. no. 65001), Bst 2.0 DNA polymerase (NEB, cat. no. M0537L), including 10× isothermal amplification buffer, dNTP mix, 25 mM each dNTP (Thermo Scientific, cat. no. R1121), T4 DNA polymerase (Thermo Scientific, cat. no. EP0062), Buffer Tango, 10× (Thermo Scientific, cat. no. BY5), T4 DNA ligase (Thermo Scientific, cat. no. EL0012), including 50% PEG-4000 solution and 10× T4 Ligation buffer and AccuPrime Pfx DNA polymerase (Life Technologies, cat. no. 12344-024), including 10× AccuPrime reaction mix. All the oligos were ordered HLPC purified (IDT).

**DNA constructs.** All DNA sequences are provided as Supplementary Table 2 and key constructs will be made available via Addgene. The HIVRT p66 domain was codon optimized for *E. coli* and synthesized (Geneart). The p51 domain was obtained by PCR as a truncated version of this gene. The murine leukemia reverse transcriptase gene is not codon optimized and was received as a gift from Prof. Timothy Lu (MIT). The HTBS part was ordered as a Gblock (IDT). The assembly of r_oligo genes into different versions of the p_O_# plasmids was performed using the Golden Gate Method[2]. Plasmid maps are provided in the supplementary Fig. 15-24. The 10bp sticky regions used for the ssDNAs to build the 4-part nanostructure were designed in the following way. The starting sequence was obtained from the original paper describing the crossover motif[3]. From this starting point, the zinc-finger sequences[4] were incorporated with random nucleotides selected by eye and changed to G or C in order to decrease the hybridization free energy. Each new sequence was tested for secondary structure using NuPack[5] and the IDT oligo analyzer software[6] and problematic sequences were mutated and retested. Using the same software, sequences were analyzed for the possibility to self-dimerize or form undesired hybridization products and potential problems were alleviated by making additional mutations.

**Detailed *In vivo* ssDNA Production Protocol**

The following is a detailed protocol for the production and purification of the 72-nt ssDNA oligo. This requires the following plasmids: pO_72, pHIV_pTp66p51 and pMLRT (all of which will be available on Addgene). This corresponds to the experiment shown in Figure 2d.

Prepare the following solution and autoclave:

- LB/Agar media- Add 12.5 grams of LB Broth, Miller (BD #244610) and 8 grams of Agar (BD #214010) to 500 mL of water. Mix and autoclave.
- Terrific Broth (TB) media- Add 50.9 grams of Terrific Broth (TEKnova # T0311) and 4 mL of glycerol to 1L of water. Mix and autoclave.
- SOB media- Add 15 grams of SOB (TEKnova # S0210) to 500 mL of water. Mix and autoclave.
- Autoclave at 250 F for 40 minutes.

Day 1 -Transformation

1. Thaw a tube of NEB 10-beta Competent *E. coli* cells (NEB) on ice for 10 minutes.
2. Pipette 50 µl of cells into a PCR tube on ice.
3. Add 1 µl containing 50-100 ng of each DNA plasmid (pO_72, pHIV_pTp66p51 and pMLRT) to the cell mixture.
4. Keep the mixture on ice for 30 minutes.
5. Heat shock the mixture at 42°C for 30 seconds.
6. Place the tube on ice for 2 minutes.
7. Prepare SOC media by adding 5 µL of 2M glucose to 500 µL SOB.
8. Pipette 500 µL of SOC into the mixture.
9. Incubate at 37°C-250 rpm for 60 minutes.
10. Centrifuge the mixed cells at 10000 *x g* for 30 sec.
11. Remove 400 �L of the supernatant and resuspend the cells in the remaining 100 �L.
12. Spread 10 µl to a selection plate containing the appropriate antibiotics (50 µg per ml spectinomycin, 25 µg per ml kanamycin and 50 µg per ml ampicillin) and incubate overnight at 37°C.

Note that steps 1, 4-6 were reproduced verbatim from NEB transformation protocol.[7]

Day 2 -

Select a colony from the plate and inoculate in 1 L of Terrific Broth (TB) containing the appropriate antibiotics (50 µg per ml spectinomycin, 25 µg per ml kanamycin and 50 µg per ml ampicillin) and 10 mM IPTG. Then, incubate the resulting solution at 37°C-250 rpm for 24 h.

Day3 - Purification of the ssDNA

After incubation, the ssDNA is purified using the TRIzol® Reagent protocol (Thermo Fisher):

1. Centrifuge 250 mL of the overnight culture in five 50 mL tubes at 5000 *x g* for 7 min at 4°C (50 mL of culture in each tube).
2. Remove the supernatant.
3. Add additional 250 mL of the remaining cells to the same five 50 mL tubes and centrifuge at 5000 *x g* for 7 min at 4°C.
4. Repeat step 2-3 until all the cells are centrifuged in the same five 50 mL tubes.
5. During centrifugation, preheat 5 mL of Max Bacterial Enhancement Reagent (Thermo Fisher) to 95°C using a heat block with 50mL conical adapter (VWR digital heatblock).
6. After centrifugation (step 1-4), resuspend the cell pellets by adding 1 mL of preheated Max Bacterial Enhancement Reagent to each 50 mL tube. Mix well and incubate the tubes at 95°C for 4 minutes.
7. Add 7 mL of TRIzol Reagent to each one of the five 50 mL tube containing the cell lysate and mix well.
8. Incubate the tubes at room temperature for 5 minutes and proceed to Phase Separation.
9. Phase Separation-During the incubation of step 8, prepare twenty-four 2-mL tubes. Add 320 μl of cold chloroform to each tube.
10. After incubation (step 8), add 1.6 mL of the TRIzol-lysate mixture to each one of the pre-prepare twenty-four 2-mL tubes each containing 320 μl of cold chloroform. Then, mix by shaking the tube vigorously by hand for 15 seconds.
11. Incubate the tubes at room temperature for 3 minutes.
12. Centrifuge the samples at 12,000 × *g* for 15 minutes at 4°C. Note that after centrifugation, the mixture separates into a lower red, phenol-chloroform phase, an interphase, and a colorless aqueous phase containing the RNA and ssDNA. The volume of the aqueous phase is ~1 mL.
13. During the centrifugation (step 12), prepare twenty-four new 2-mL tubes. Add 1 mL of cold isopropanol to each tube.
14. After the centrifugation of step 12, transfer carefully the 1 mL resulting colorless aqueous phase from each tube to one of the pre-prepared twenty-four 2-mL tubes each containing 1 mL of cold isopropanol (step13). Note that each tube should contained 1 mL of colorless aqueous phase and 1 mL of cold isopropanol. Then, mix by inverting the tubes.
15. Incubate the tubes at room temperature for 10 minutes.
16. Centrifuge at 15,000 × *g* for 10 minutes at 4°C.
17. Remove the supernatant carefully without disturbing the pellet (a gel-like pellet formed at the side and bottom of the tube).
18. Resuspend the pellet in 1 mL 75% ethanol. Mix well by vortexing.
19. Centrifuge at 7500 × *g* for 5 minutes at 4°C. Discard the supernatant.
20. Air-dry the pellet for 30 minutes at room temperature.
21. Resuspend the pellet from each twenty-four tubes with 50 μL water by pipetting up and down.
22. Collect the twenty-four 50 μL solutions to a single new 2-mL tube ~1.2 mL and add 1.2 μl of RNAse A (100 mg per ml, Qiagen).
23. Incubate the resulting solution from step 22 at 37°C for overnight to allow the RNA degradation.

Note that steps 12, 15-19 were reproduced verbatim from TRIzol® Reagent protocol (Thermo Fisher).[8]

Day 4-Concentration and PAGE Analysis

The 1.2 mL ssDNA solution is then cleaned and concentrated using the oligo clean and concentrator kit (ZYMO Research CORP.). Note that the original provided Zymo-Spin™ IC columns have been replaced with the Zymo-Spin™ IIC Columns (Zymo-Spin™ IIC Columns have an higher DNA binding efficiency):

1. Add 2.4 mL of Oligo Binding Buffer to the resulting solution form the purification section (step 23).
2. Add 9.6 mL of ethanol (95-100%), mix briefly by pipetting and transfer the mixture to twenty-four Zymo-Spin™ IIC Columns.
3. Centrifuge at 12,000 x *g* for 30 seconds. Discard the flow-through.
4. Add 750 µl DNA Wash Buffer to each column, centrifuge at 12,000 x *g* for 30 seconds and discard the flow-through.
5. Centrifuge the empty columns at maximum speed for 1 minute.
6. Elute the ssDNA by adding 35 l of water to one of the twenty-four columns matrix and centrifuge at 12,000 x *g* for 30 seconds in a clean collection tube.
7. Collect the resulting solution (if the volume is lower than 35 l adjust by adding water).
8. Elute the ssDNA from another column by adding the 35 l resulting solution from step 7 to the column matrix and centrifuge at 12,000 x *g* for 30 seconds in a clean collection tube.
9. Repeat step 7-8 until the entire ssDNA have been eluted from all of the twenty-four columns using the same 35 l solution.

Note that steps 3-5 were reproduced verbatim from the oligo clean and concentrator kit (ZYMO Research CORP.).[9]

PAGE Analysis

The purified ssDNA solution is then run on a 15% Mini-PROTEAN® TBE-Urea Gel (Precast Gel #4566053, BI0-RAD) in a Tris-borate-EDTA (TBE) buffer solution, that included Tris base (89 mM, pH=7.9), boric acid (89 mM) and EDTA (2 mM):

1. Prepare the sample by diluting 10-time the resulting ssDNA from *“cleaned and concentrated”* section (1 L of ssDNA with 9 L of water).
2. Add 10 L of loading dye and incubate for 5 min at 95°C.
3. Load the sample in the well of the gel.
4. Run the gel on a Mini-PROTEAN Tetra Cell (BI0-RAD #165-8000) under a constant voltage (200 V) for 40 minutes.
5. After electrophoresis, stain the gel with SYBR Gold nucleic acid gel stain (Invitrogen) and image.

Note that additional samples are prepared and run in parallel containing the same 72-nt ssDNA sequence and different lengths of oligos from IDT that are used as an artificial ladder:

1. Prepare 10 L of the IDT samples containing 30 nM of ssDNA oligo.
2. Add 10 L of loading dye to each samples and incubate for 5 min at 95°C.
3. Load and run the samples in the same gel.

**Supplementary References:**


1. 1. Ying-Ja, C. *et al.* Characterization of 582 natural and synthetic terminators and quantification of their design constraints. *Nat. Methods* 10, 659–664 (2013).
2. Engler, C. , Kandzia, R. , Marillonnet, S. A one pot, one step, precision cloning method with high throughput capability. PLoS ONE doi:10.1371/journal.pone.0003647 (2008).
3. Fu, T. J. , Seeman, N. C. DNA Double-crossover Molecules. *Biochemistry* **32**, 3211–3220 (1993).
4. Conrado, R. J. *et al* DNA-guided assembly of biosynthetic pathways promotes improved catalytic efficiency. *Nucl. Acids Res.* **40**, 1879-1889 (2012).
5. Zadeh, J.N *et al* NUPACK: analysis and design of nucleic acid systems. J Comput Chem, 32, 170–173, 2011.
6. PrimerQuest® program, IDT, Coralville, USA. Retrieved 12 December, 2012. http://www.idtdna.com/Scitools.
7. www.neb.com/protocols/1/01/01/high-efficiency-transformation-protocol-c3019a
8. www.tools.thermofisher.com/content/sfs/manuals/trizolmax_man.pdf
9. www.zymoresearch.com/downloads/dl/file/id/480/d4060i.pdf
